# Longitudinal Antigenic Sequences and Sites from Intra-Host Evolution (LASSIE) Identifies Immune-Selected HIV Variants

**DOI:** 10.3390/v7102881

**Published:** 2015-10-21

**Authors:** Peter Hraber, Bette Korber, Kshitij Wagh, Elena E. Giorgi, Tanmoy Bhattacharya, S. Gnanakaran, Alan S. Lapedes, Gerald H. Learn, Edward F. Kreider, Yingying Li, George M. Shaw, Beatrice H. Hahn, David C. Montefiori, S. Munir Alam, Mattia Bonsignori, M. Anthony Moody, Hua-Xin Liao, Feng Gao, Barton F. Haynes

**Affiliations:** 1 Los Alamos National Laboratory, Los Alamos, NM 87545, USA; kshitij@lanl.gov (K.W.); egiorgi@lanl.gov (E.E.G.); tanmoy@lanl.gov (T.B.); gnana@lanl.gov (S.G.); asl@lanl.gov (A.S.L.); 2 Santa Fe Institute, Santa Fe, NM 87501, USA; 3 Perelman School of Medicine, University of Pennsylvania, Philadelphia, PA 19104, USA; glearn@mail.med.upenn.edu (G.H.L.); fkreider@mail.med.upenn.edu (E.F.K.); yingyl@mail.med.upenn.edu (Y.L.); shawg@upenn.edu (G.M.S.); bhahn@upenn.edu (B.H.H.); 4 Duke Human Vaccine Institute, Duke University Medical Center, Durham, NC 27710, USA; monte@duke.edu (D.C.M.); alam0004@mc.duke.edu (S.M.A); mattia.bonsignori@duke.edu (M.B.); tony.moody@duke.edu (M.A.M.); hliao@duke.edu (H.-X.L.); feng.gao@duke.edu (F.G.); hayne002@mc.duke.edu (B.H.H.)

**Keywords:** human immunodeficiency virus type 1, vaccine, neutralizing antibodies, immunogen design, envelope glycoprotein, coevolution, immune escape, quasispecies, antigenic swarm, selection

## Abstract

Within-host genetic sequencing from samples collected over time provides a dynamic view of how viruses evade host immunity. Immune-driven mutations might stimulate neutralization breadth by selecting antibodies adapted to cycles of immune escape that generate within-subject epitope diversity. Comprehensive identification of immune-escape mutations is experimentally and computationally challenging. With current technology, many more viral sequences can readily be obtained than can be tested for binding and neutralization, making down-selection necessary. Typically, this is done manually, by picking variants that represent different time-points and branches on a phylogenetic tree. Such strategies are likely to miss many relevant mutations and combinations of mutations, and to be redundant for other mutations. Longitudinal Antigenic Sequences and Sites from Intrahost Evolution (LASSIE) uses transmitted founder loss to identify virus “hot-spots” under putative immune selection and chooses sequences that represent recurrent mutations in selected sites. LASSIE favors earliest sequences in which mutations arise. With well-characterized longitudinal Env sequences, we confirmed selected sites were concentrated in antibody contacts and selected sequences represented diverse antigenic phenotypes. Practical applications include rapidly identifying immune targets under selective pressure within a subject, selecting minimal sets of reagents for immunological assays that characterize evolving antibody responses, and for immunogens in polyvalent “cocktail” vaccines.

## 1. Introduction

It is not yet known how to elicit protective immunity against HIV-1, and neutralizing antibody induction remains a central focus of HIV vaccine research. Neutralizing antibodies are immune correlates of protection in all antiviral vaccines licensed to date [1,2]. Administration of neutralizing antibodies can confer protection in SHIV challenge models with rhesus macaques [3,4]. During the natural course of HIV infection, typically a single transmitted-founder (TF) virus establishes infection [5,6]. The virus population grows exponentially, with random mutations that initially accumulate following a Poisson distribution of inter-sequence distances [5,6]. The viral load eventually declines and resolves to a quasistationary set-point [7], influenced by both host and viral factors [8]. HIV-1 is maintained as a continuously evolving quasispecies population throughout chronic infection [9], with diversification driven by adaptive immune responses, including antibody [10,11,12,13,14,15,16,17,18] and T cell responses [19,20,21]. Mutations that facilitate immune evasion are positively selected and become more frequent, while mutations that result in a relative fitness disadvantage do not persist. Though neutral mutations may also drift to higher frequency with rates that depend on the effective population size, positive selection exceeds drift at driving envelope evolution within hosts [22,23,24].

During the chronic phase of infection, fifty percent of chronically HIV-1 infected individuals’ antibody responses cross-neutralize 50% of HIV-1 primary isolates and breadth of neutralization responses varies uniformly across individuals, from neutralization of only a few heterologous viruses, to sera with great breadth and potency [17]. Plasma samples from individuals with the most potent and broad antibody neutralization are frequently singled out for detailed study [25,26,27,28]. Such studies include investigations of both viral and B cell lineages to understand the immunological processes that elicit effective neutralization responses and to inform strategies for vaccine design [15,16,18,29,30,31]. In general, autologous strain-specific neutralizing antibodies (nAbs) begin to develop within the initial months after infection, and rapidly select for viral escape variants [11,14]. High titers of more broadly neutralizing antibodies (bnAbs) develop in a subset of cases, but only after years of infection, and bnAb development is associated with persistently high levels of viral replication [32,33]. Previous longitudinal studies of HIV-1 infected individuals have shown that viral diversification precedes the acquisition of breadth, which suggests that antigenic diversity may be necessary for bnAb induction *in vivo* [15,18]. Several antibody lineages can place selective pressure on the same epitope at the same time, and escape from one antibody lineage can enhance recognition by another lineage, in a delicate evolutionary balance [16].

Viruses in individuals with bnAbs characterized to date have escaped from otherwise broadly reactive neutralizing antibody responses [34]. Antibodies that recapitulate much of the potency and breadth of polyclonal sera have been isolated from subjects with high bnAb titers [35,36]. The developmental pathway of B cell immunoglobulin genes from the initial triggering of an HIV-1 specific clonal lineage, through the acquisition of potency and breadth later in infection, is an active research frontier. Properties of evolving viral Env proteins that stimulate or facilitate the important transition from autologous to heterologous reactivity are only beginning to be understood [15,18]. Understanding the events in natural HIV-1 infection that result in broader humoral responses should ultimately enable new vaccine strategies to elicit potent, broadly cross-reactive nAbs. Thus, a continuing research priority has been to characterize virus coevolution with antibodies in individuals who develop the greatest potency and breadth of neutralization [15,16,18,31,37,38]. Working back from mature bnAb clonal lineages, through ancestral intermediates, to the unmutated germline precursor, has begun to help define the process of bnAb development [15,18,29,30,35,37,38,39,40]. To study antibody/viral coevolution, analysis of longitudinally (*i.e.*, serially) sampled sequences, which represent both the antibody population as it undergoes affinity maturation and the virus population as it evolves to evade the ongoing immune responses, explores mutational patterns that are selected over time [15,18].

Here we describe a new computational approach, called LASSIE, a two-step process to conduct longitudinal sequence analyses and inform reagent design. The first part of the LASSIE approach allows one to define and visualize sites that are potentially under positive selective pressure in the viral population. Identifying sites under putative immune selection can immediately help guide inference of antibody specificities that are active in the plasma, enable tracking of their appearance through time, and identify key mutations to be characterized during experimental follow-up studies. The second part of the approach is an algorithm that objectively down-selects sequences from a much larger sequence sample, yielding a subset of viral variants that represents mutations among the selected sites. We call the resulting subset of sequences an “antigenic swarm”, which captures mutations at sites that are under the most potent selective pressure as they first emerge in the evolving HIV-1 quasispecies [15,18,31]. The size of the sequence subset involves a trade-off between the experimental cost of including more variants and the degree of selection to be represented. LASSIE involves two parameters that can be adjusted to balance these factors, explore the data, and choose the most representative set given experimental feasibility and sample-size limitations. To validate LASSIE, we retrospectively analyzed the Env quasispecies complexity in an African HIV-infected individual (CH505), for which we already have extensive information regarding antibody interactions and targeted Env epitopes [15,16]. This approach allowed us to determine how well the relevant diversity was captured by our computational method.

LASSIE can be used to select Envs (or similarly diversifying proteins) for expression and binding studies, or to generate pseudoviruses for use in neutralization assays. In turn, the resulting reagents can be used to study relationships between viral phenotype and genotype, and to investigate in detail how neutralizing antibody responses develop by affinity maturation. Moreover, the chosen Envs can be used as experimental polyvalent “swarm” HIV vaccine immunogens.

## 2. Results

**Figure 1:**
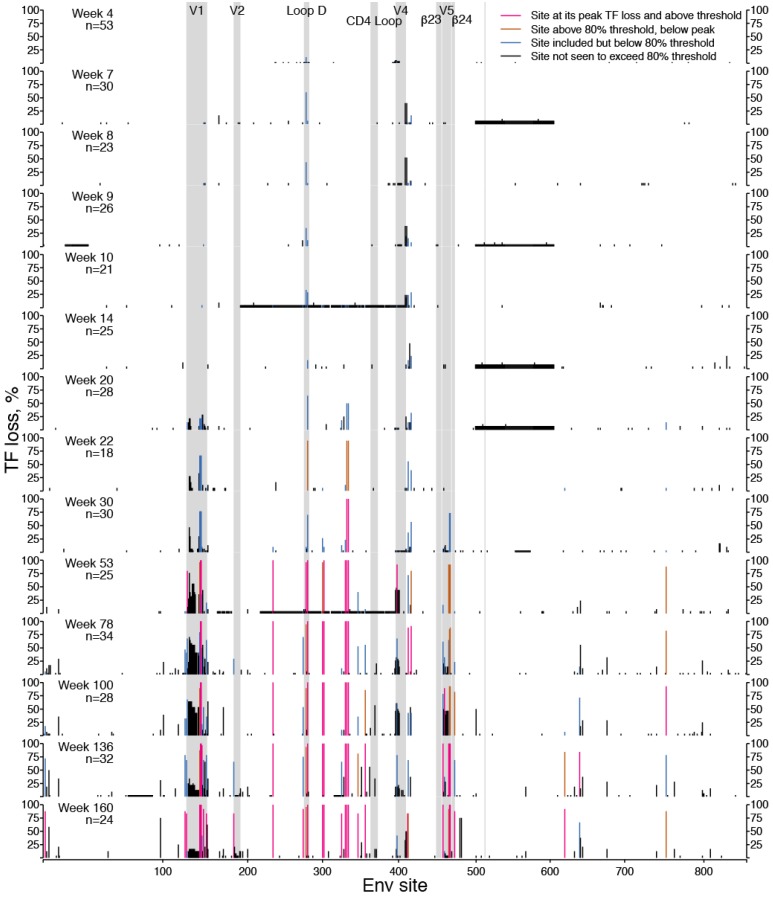
Loss of ancestral transmitted-founder (TF) amino acids in Envs from CH505. For 953 aligned Env sites spanning the full-length protein, TF loss is proportion of non-TF mutations per time-point sampled from the study participant CH505. TF loss is computed for each of 14 time-points sampled longitudinally, weeks 4 through 160, with the number of Envs sequenced (*n*) per time-point as shown. Bar colors vary over time to indicate 35 sites with at least 80% TF loss in any time-point, whether at peak TF loss (pink), below peak but above the 80% cutoff (brown), or below 80% (blue). Variable sites that were not selected for further consideration, because they never exceeded 80% TF loss in ant time-point during the study period, are also depicted (black bars). Grey boxes identify variable loops, which contain hypervariable regions that evolve by insertion and deletion, and other gp120 landmarks. A thin grey line marks the boundary between gp120 and gp41. The time of the sample is shown as weeks post infection. Env site numbers indicate HXB2 positions in the CH505 protein alignment, beginning with the signal peptide start codon in column one and ending with the stop codon at position 857.

Building upon recent insights into antibody/Env coevolution in individual CH505 [15,16,41], we first applied LASSIE to this subject as a test case, to determine how well the method performed in a situation where key epitopes had already been defined and characterized. We aligned 397 Env sequences from 14 time-points sampled over three years across 953 Env sites and computed TF loss (Figure 1). Alignment to the HXB2 reference sequences provided a standardized numbering scheme. Two sequences from CH505 (KM284795 and KC247403) contain aberrant in-frame insertions (29 amino acids after HXB2 position 33 and 38 amino acids after site 86, respectively) which, in addition to the variable-length loop regions, disrupted the x-axis linear scaling in Figure 1. Ten other sequences contained long, in-frame deletions, leaving 385 full-length Envs for analysis. The excluded sequences appear as long, low bars in Figure 1.

### 2.1. Site Selection

Figure 1 shows TF loss per site for each time-point sampled, from week 4 through week 160 post-infection. Clearly, most sites show little or no TF loss. Sites with high levels of TF loss are putative escape mutants due to immune selection. Because we counted an insertion or deletion relative to the TF virus as a change, the hypervariable V1, V2, V4, and V5 regions also showed TF loss, largely due to length variation. We used TF loss to list sites where frequency of the TF form fell below a fixed cutoff percentage. The cutoff is a parameter that can be adjusted as needed; here we used the value of 80% TF loss in samples from at least one time-point. We defined “peak” TF loss per site as the highest TF loss in that site over all time-points sampled (often the “peak” was maintained over many samples), and used it to select candidates for sites under immune selection.

#### 2.1.1. TF Loss Varied across Sites

**Figure 2:**
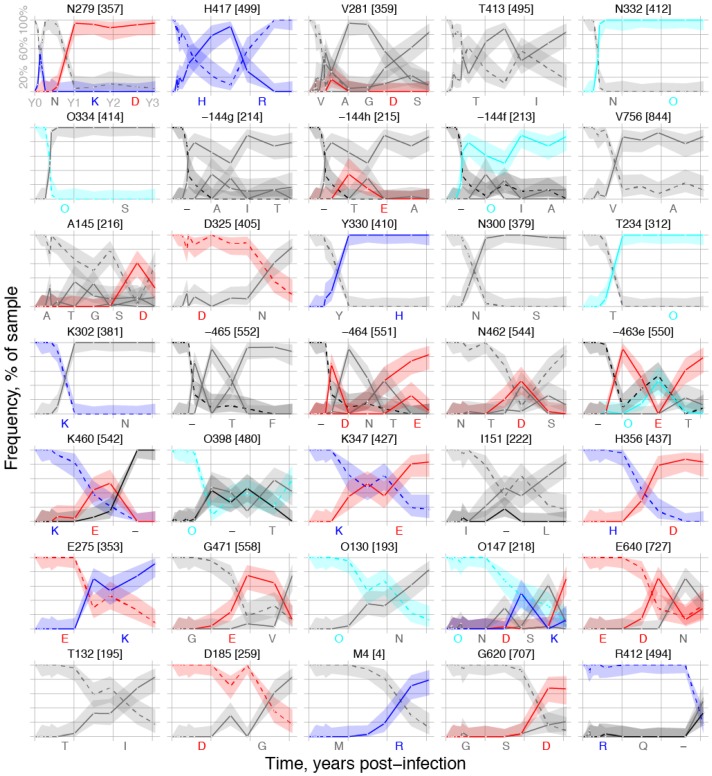
Variant frequency dynamics within sites. The single TF virus amino acid (dashed lines with 100% initial frequency) yields to putative escape mutations (solid lines with 0% initial frequency) over the sampling period. Letters below each plot list mutations in order of appearance, with panels ordered by timing of TF loss. Numbers above each plot denote TF form, HXB2 position, and alignment column, e.g., “N279 [357] indicates loss of the transmitted asparagine at HXB2 position 279, alignment column 357. Lower-case letters denote insertions at the C-terminal end of the HXB2 site given. Colors indicate positive (blue) and negative (red) charges and an “O” is used instead of “N” to indicate a potentially glycosylated asparagine (cyan), *i.e.*, an N that is embedded in a glycosylation motif of Nx[ST], where x can be any amino acid except Pro, followed by either Ser or Thr. Vertical bars indicate the sampling year, abbreviated in the upper left panel as Y0, Y1 and Y2, with a single TF virus starting at Y0, then followed by the first time-point sampled, estimated to be 28 days post infection for CH505 [15]. Shaded regions show 95% confidence intervals for variant frequencies, computed from the binomial probability distribution, given the number of sequences sampled per time-point. Several distinct insertions arise after HXB2 position 144 in the V1 loop, shown in panels in the middle of the second row as 144f, 144g, and 144h. Lower-case letters (f through h) specify the relative positions of the inserted region in the alignment [45]. The TF lacks an amino acid in this position, which is characterized as a gapped state and represented by a dash (–) to maintain the alignment. Over time, new and distinct insertions arise that span this position, with major and minor variants carried along with distinctive insertions occurring at positions 144f, 144g, and 144h (e.g., in 144 g, three different insertions are maintained, which include A, I and T).

Initially dominated by the TF form, the quasispecies acquired mutational variants over time, displaying different dynamics among sites with high TF loss. Figure 2 depicts variant frequencies in subject CH505 over time in the 35 sites, which had over 80% TF loss in at least one time-point. The rate of TF loss was slower in some sites than in others. Such slow transitions could reflect the evolving immune response and newly arising selective pressure. In qualitative terms, we saw four dynamic categories, designated *i*-*iv*. First (*i*), some sites showed complete replacement of the TF form with another single variant, whether fast or slow, e.g., the shift of a glycosylation site from position 334 to 332 (top-right panel “N332” in Figure 2, N→O, and O334 in the next row, from O→S, where “O” is an asparagine embedded in a glycosylation motif, as described in Section 4.2). Next (*ii*), in some sites, the initial TF replacement was followed by one or more additional mutations arising sequentially. For example, site 279, located in Loop D, was initially an asparagine, but a transient lysine mutation yielded to an aspartic acid after transient reversion to the TF asparagine (Figure 2, top-left, N→K→N→D). Third (*iii*), some sites reverted to the TF form after high TF loss. For example, site 417, initially histidine, was predominantly an arginine from about six months to nearly two years after infection, but then reverts to the ancestral histidine (Figure 2, top row, second panel, H→R→H). Finally (*iv*), some sites exhibited sustained polymorphisms. These were particularly common in hypervariable loops, where insertions and deletions are predominant evolutionary processes, and distinct subpopulations may carry divergent forms. For example, distinct insertions arise in V1 after HXB2 position 144, maintained at different frequencies (Figure 2, middle of the second row, 144 g, 144h, and 144i). Appearance of such patterns in hypervariable regions may be alignment dependent, and should be reviewed in the context of the full alignment to ensure they represent distinct and common forms in the viral quasispecies, rather than alignment artifacts.

We sampled a median of 25 (range 18–53) sequences across 14 time-points, and sampling 18 sequences, our minimum, corresponds to an 85% probability of detecting variants prevalent at 10% or more in the sample. Figure 2 also shows estimated 95% confidence intervals for variant frequencies. For clarity, the mutations that never attain 15% frequency in any sample are not shown.

Given our sampling constraints, we could estimate relative frequencies of the different mutational classifications described above. Simple shifts, like (*i*), were the most common form of TF loss, evident in 16 of the 35 selected sites (46%). Serial mutations, like (*ii*), were also common and could be the direct result of serial escape, due to new pressures imposed by adaptation of the evolving antibody response to an initial escape mutation, driving continued selection. Alternatively, serial replacements could result from complex interactions with multiple antibodies in a polyclonal response [16], or pressures resulting from balancing fitness costs and/or compensatory mutations in a changing evolutionary milieu. Transient losses (*iii*) reverting to the TF form were rare, and clear restoration of the TF as the dominant form after a loss of at least 80% occurred in only 2 of 35 positions (6%), positions 417 and 462 (Figure 2). Position 279 had a transient reversion to the TF form, and position 145 may have been reverting at the last time-point sampled. Different underlying reasons for this pattern could be at play, such as a fitness cost for a mutation that was carried along with a neighboring mutation, or a changing immunological environment in the host, which could transiently favor a mutation with a modest fitness cost [14,42,43,44].

#### 2.1.2. Peak TF Loss Identified Selected Sites

**Figure 3:**
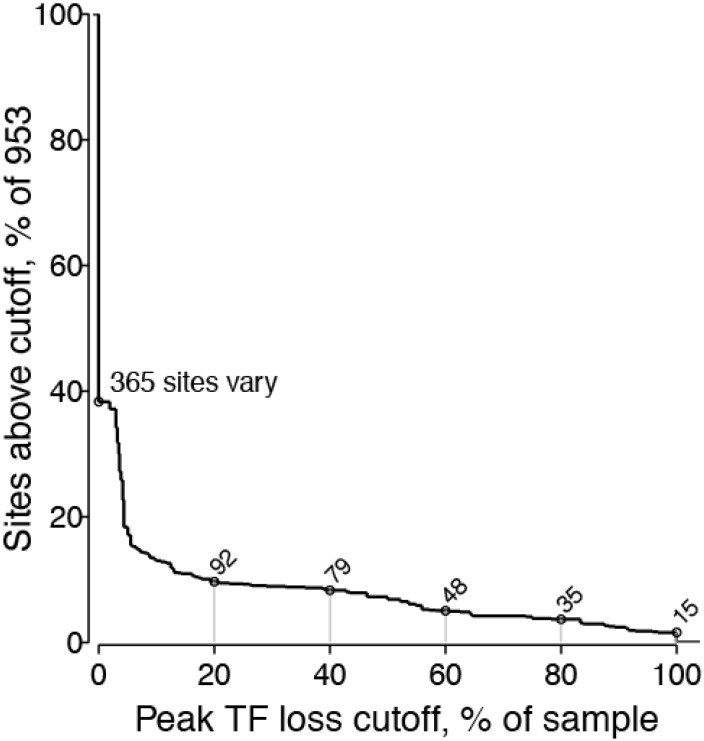
Cumulative distribution of peak TF loss over 953 aligned Env sites. Peak TF loss is the greatest proportion of non-TF variants in any time-point sampled, which corresponds to the minimum for each dashed line in Figure 2. Of 953 aligned sites, 365 (38.3%) varied and the others did not vary among sequences sampled throughout the period studied. We selected 35 sites with at least 80% peak TF loss for further study. Other cutoff values would yield more (e.g., 48 at 60% TF loss) or fewer sites (e.g., 15 at 100% loss) for consideration.

Together, 15 sites completely lost the TF form during the three-year sampling period, while the other 938 aligned sites never reached 100% TF loss. The cumulative distribution of peak TF loss per site (Figure 3) indicated that 588 of 953 sites (62%) were strictly invariant, and 64 (6.7%) lost over 50% TF. We selected the 35 sites with at least 80% peak TF loss for further study and Env selection. The 80% cutoff is the threshold we chose to use for this presentation of these data. Increasing the TF loss cutoff decreases the number of sites selected, and working with other cutoff values can adjust the number of selected sites for subsequent investigation. This value can be chosen in light of available resources.

#### 2.1.3. Selected Sites were Consistent with Antibody-Driven Selection

The time at which TF loss started to emerge at each selected site in the sampled virus population naturally varied from one site to another (Figure 1 and Figure 2). The cumulative amount of TF loss also varied. Cumulative TF loss over time had a simple geometric interpretation as the area above the dashed TF line in the plots of frequency over time that appeared in Figure 2.

Table 1 lists the 35 selected sites with at least 80% peak TF loss, with rank determined by the earliest time at which any non-TF variant exceeded 10%, and ties resolved by cumulative TF loss sorted in descending order. Most (91%) of the selected sites occurred in gp120.

**Figure 4:**
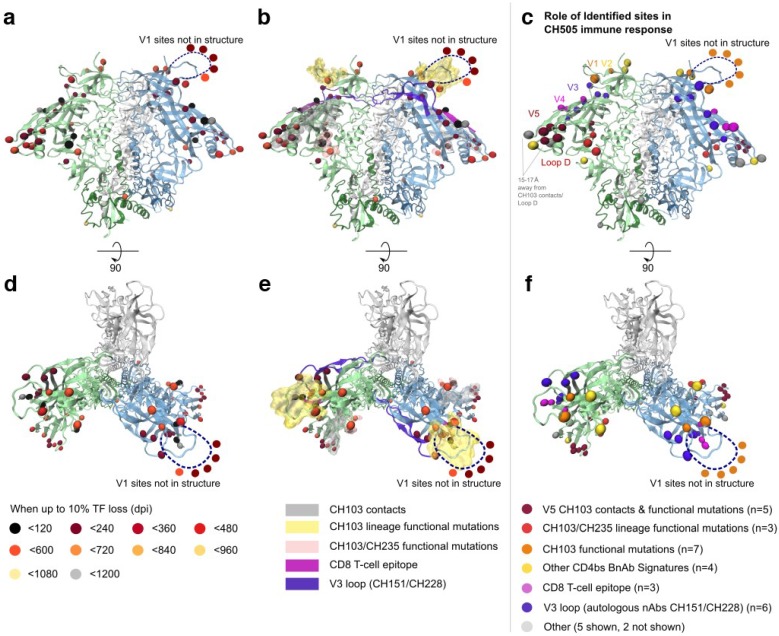
Selected sites are localized to the known immunogenic regions in CH505, as visualized by mapping onto the structure of the engineered BG505 SOSIP trimer (Protein Data Bank ID 4TVP [48]). Selected sites are depicted as spheres, colored to indicate the timing of their emergence. (**a**) Side view, oriented with viral membrane towards bottom; (**b**) Additional colored highlights indicate known immunogenic regions; (**c**) Selected sites are colored to show which immune pressures are known to have induced TF loss; (**d**–**f**) The corresponding details from top view, as seen from host cell membrane. Table 1 lists symbol colors for each selected site.

In the context of the Env trimer structure, the selected sites formed three localized clusters on the outer domain of gp120 (Figure 4). The clustered patches of selected sites on gp120 corresponded to the three known regions of immunological pressure in individual CH505. The first cluster of three selected mutations (HXB2 positions 412, 413, 417) was in a CTL epitope, which was targeted early in infection in CH505 and conferred early CTL escape [16]. The second cluster of six selected sites (300, 302, 325, 330, 332, 334) was located within the V3 loop, or in the glycosylation site at its base. Two autologous neutralizing anti-V3 antibodies, DH151 and DH228, were isolated from CH505. These antibodies could only neutralize heterologous Tier 1 viruses at the heterologous population level, but potently neutralized a subset of autologous CH505 Tier 2 viruses, shown by linear peptide array binding to target a linear epitope in the V3 loop, which encompass the V3 loop sites in this second cluster [41]. Thus, this second cluster of V3 sites may be relevant to escape from members of this nAb lineage.

**Table 1:** Selected sites. Summary of the extent of TF loss and timing of the mutation in the subject. These CH505 Env sites showed at least 80% TF loss in at least one time-point.

	HXB2 Site	Peak Loss	When Up	Rank	MEME *p*-Value	Immune Pressure	Notes
	4	87.5	d701	33	NA ^1^	NA	Signal peptide
∙^2^	130	87.5	d547	28	0.086	CD4bs	PNG site at base of V1, near VRC01 contact [46]
∙	132	83.3	d547	31	* ^3^	CD4bs	V1 indels cause CH103 resistance [16]
∙	144f	100	d141	9	*	CD4bs	V1 indels cause CH103 resistance [16]
∙	144g	100	d141	7	*	CD4bs	V1 indels cause CH103 resistance [16]
∙	144h	100	d141	8	*	CD4bs	V1 indels cause CH103 resistance [16]
∙	145	96.8	d141	11	*	CD4bs	V1 indels cause CH103 resistance [16]
∙	147	91.7	d547	29	*	CD4bs	V1 indels cause CH103 resistance [16]
∙	151	83.3	d371	24	*	CD4bs	V1 indels cause CH103 resistance [16]
∙	185	83.3	d547	32	0.013	CD4bs	Signature site for CD4bs bnAb b12 [47]
∙	234	100	d211	15	NA	CD4bs	Signature site for CD4bs VRC01 & NIH45-56 [47]
∙	275	91.7	d547	26	0.044	CD4bs	Loop D, CH103 contact, CH235 resistant [15,16]
∙	279	95.8	d28	1	0.090	CD4bs	Loop D, CH235 resistant, CH103 sensitive [15,16]
∙	281	100	d64	3	**0.00007 **^4^	CD4bs	Loop D, CH235 resistant, CH103 sensitive [15,16]
∙	300	100	d211	14	NA	V3 loop	V3 autologous nAb in CH505 [41]
∙	302	100	d211	16	NA	V3 loop	V3 autologous nAb in CH505 [41]
∙	325	83.3	d141	12	NA	V3 loop	V3 autologous nAb in CH505 [41]
∙	330	100	d157	13	NA	V3 loop	V3 autologous nAb in CH505 [41]
∙	332	100	d141	5		V3 loop	V3 autologous nAb in CH505 [41]
∙	334	100	d141	6	0.029	V3 loop	V3 autologous nAb in CH505 [41]
∙	347	83.3	d371	23	0.0074	CD4bs	15–17 Angstroms from CH103 contacts
∙	356	100	d547	25	0.018	CD4bs	Adjacent to CD4bs bnAb 12A12 signature [47]
∙	398	91.3	d371	22	0.0088	CD4bs	15-17 Angstroms from CH103 contacts
∙	412	83.3	d1121	35	NA	CTL	CTL epitope V4 loop [16]
∙	413	88.2	d64	4	**0.00004**	CTL	CTL epitope V4 loop [16]
∙	417	91.2	d51	2	**0.00073**	CTL/CD4bs	CTL epitope V4 loop; CD4bs b12 contact [16]
∙	460	100	d371	21	*	CD4bs	V5, CH103 contact region, resistance [15,16]
∙	462	89.3	d211	19	*	CD4bs	V5, CH103 contact region, resistance [15,16]
∙	463e	100	d371	20	*	CD4bs	V5, CH103 contact region, resistance [15,16]
∙	464	100	d211	18	*	CD4bs	V5, CH103 contact region, resistance [15,16]
∙	465	100	d211	17	*	CD4bs	V5, CH103 contact region, resistance [15,16]
∙	471	87.5	d547	27	0.0057	CD4bs	CH103 contact [16]
∙	620	91.7	d953	34	**0.0026**	NA	gp41
∙	640	83.9	d547	30	0.0054	NA	gp41
∙	756	92.9	d141	10	0.0035	NA	gp41 cytoplasmic tail

^1^ “NA” indicates MEME found no associations with positive selection having *p* below 0.1.

^2^ Symbol color indicates known immune pressures in CH505 that likely drove selection (Figure 4c,f).

^3^ Asterisks (*) in this column indicate a site in hypervariable regions of V1 or V5, where evolution is enhanced by insertions and deletions. Because this mode of evolution fails to satisfy assumptions of the statistical model, the *p*-values (false-positive rates) are not readily interpreted. Regardless, MEME found support for positive selection in these regions.

^4^ Bold text indicates MEME *q*-values (false-discovery rates) below 0.2.

The third cluster, in the CD4 binding site (CD4bs), is the most complex. The CD4bs is the target of both the CH103 bnAb lineage [15] and the CH235 nAb cooperative lineage [16] in subject CH505. Many of the 32 selected gp120 sites included structurally defined contacts for CD4 [46,49], and several previously studied CD4bs bnAbs, including VRC01 [46,49], NIH45–46 [50], and b12 [51] (Table 1). Although the current study is retrospective, this pattern of mutations indicates the presence of CD4bs antibodies in the subject, even prior to isolation of CH235 and related nAbs at week 41 [16], as this region was apparently under selective pressure very early, particularly in the loop D region [52]. As expected, CH103 contacts and previously characterized resistance mutations were well represented among the selected sites [16]. Three selected sites (279, 281, 275) were localized to CH103 light-chain contacts near loop D (Figure 4), a region that rapidly accumulated mutations as a result of escape from the autologous CD4bs neutralizing antibody CH235; these mutations rendered the virus more susceptible to the CH103 early lineage members. Six CH103 heavy-chain contacts in and near V5 (460, 462–465, 471) were also among the selected sites, and mutations in this region conferred CH103 resistance [53]. V1 loop mutations also conferred CH103 resistance, and seven sites in V1 were among the 35 selected sites (132, 144f, 144g, 144h, 145, 147, and 151). Three of these were inserted together in V1 after position 144. Finally, five additional selected sites are known to be important for other CD4bs bnAb interactions, providing indirect evidence that they may be important for either CH103 or CH235, or both. These are: 417, a contact for the CD4bs bnAb b12 [51], and 185, a V2 region signature site for b12 [47]; 234, a signature site for CD4bs bnAbs VRC01 and NIH45-46 that is near Loop D [47]; the glycosylation site N130, adjacent to a VRC01 contact [46]; and position 356, adjacent to a 12A12 signature [47]. We identified selected sites that were relevant to the other antibodies noted in this section using the Los Alamos HIV-database genome browser [54] and CATNAP tool [55].

**Figure 5:**
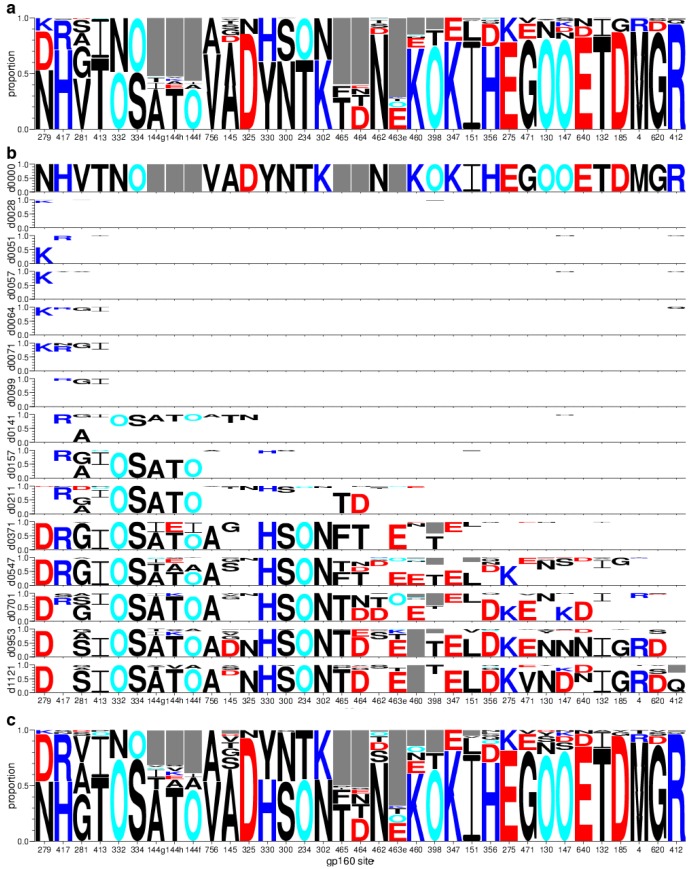
Variant frequency across 35 sites selected from CH505 Env gp160. (a) Population variant frequencies, computed from 385 aligned, full-length protein sequences; (b) Temporal development of variant frequencies. To emphasize TF loss progression, frequency of the TF form below the first row is blank. Each row corresponds to one time-point sampled for the three-year study period, days 0–1121 (d0000 through d1121); (c) Variant frequencies in swarm set of 54 selected Envs. Symbol height is proportional to amino acid frequency per site. Colors correspond to Figure 2. The gaps inserted to maintain the alignment appear as grey boxes to represent indels. Site order follows ranks listed in Table 1. This visualization was produced from modified sequence logos by the lassie package; see Section 4.4 for availability.

Together, 29 of the 35 selected sites (83%) are related to the three epitopes that were functionally defined in this subject during the time period under study, despite these sites being simply and objectively identified based solely on the TF loss criterion (Table 1). Of the six sites that were not directly related, three were gp41 sites (620, 640, 756) and one was in the signal peptide (position 4). The other two (398 and 347) were clustered near position 356 in gp120, and both were near but not in the CH103 contact region (indicated in Figure 5c as 15–17 Angstroms away from CH103 contacts). These six mutations in sites selected by TF loss are indirectly implicated as having biological or immunological significance, and may suggest strong leads for follow-up experimental investigation.

#### 2.1.4. Comparison of Selected Sites Identified by LASSIE and Phylogenetic Methods

MEME is an analysis method that identifies sites under episodic diversifying selection within a phylogenetic reconstruction [56], and represents a class of tools that identifies positive selection by comparing relative levels of synonymous and non-synonymous mutations in the dataset. While such tools are very useful, their application comes with several caveats. Here we compare the sites identified by the two methods, MEME and LASSIE, which correspond to several dynamic categories. Using LASSIE, we identified 35 sites with the TF loss criterion, while MEME identified 14 sites of highest interest, with a *q*-value (false-discovery rate) below 0.2.

Both methods identified multiple sites in the V1 and V5 hypervariable regions, eight sites with LASSIE and nine sites with MEME. Identifying these regions as foci of positive selection is appropriate, because changes in both V1 and V5 have been shown to confer distinct immunological phenotypes with respect to the evolving nAb lineages in subject CH505 [15,16,53]. However, a concern for interpreting the MEME results is that the model used to compute positive selection probabilities assumes that codons evolve by base substitutions. In hypervariable regions, this assumption is violated, and frequent insertions and deletions dominate the mutational framework. This is readily evident in the full alignment, but to illustrate it further, Tables S1 and S2 (in the Supplementary file) list each distinct variant form of the V1 and V5 hypervariable regions sampled from subject CH505. This shows clearly the effects of insertions and deletions on the evolution of these regions. MEME identified some of these positions, as it should, but the *p*- and *q*-values are not readily interpreted in the context of the evolutionary model. LASSIE also identifies V1 and V5 hypervariable regions as candidates for positive selection, simply by counting changes from the transmitted-founder state over time.

Outside of the V1 and V5 hypervariable regions, MEME identified an additional site with a *q*-value below 0.2, which LASSIE did not identify. This site, HXB2 position 411 (codon 493) was in the known CTL epitope, HXB2 positions 409–418 (codons 491–500) [16], which may confer CTL resistance. The TF frequency for this site never falls below 40% in a time-point. While this site is likely driven by immune selection, incomplete resistance or viral fitness costs associated with escape mutations at this site presumably make other local escape mutations more competitive. Regardless, this site may be of interest, and with LASSIE one could include it with the list of sites for consideration during the sequence-selection phase of analysis, if desired. An additional 14 sites identified by LASSIE were also found by MEME with a more inclusive *p*-value (false-positive rate) cutoff of 0.1, meaning they did not withstand the correction for multiple tests in MEME, but were identified as interesting by both.

A class of sites likely to be of biological interest is those captured by LASSIE and missed by MEME. These sites in CH505 are HXB2 positions 4, 234, 300, 302, 325, 330, and 412 (Table 1 and Figure 2) and are illustrated in Figure S1 to show the TF loss pattern clearly. These sites generally have either a single mutation (or very few) among internal branches of a phylogenetic reconstruction, and cannot be statistically validated using an approach like MEME. However, once such a mutation emerges, the TF form is essentially lost, and replaced by the mutation, a selective sweep. LASSIE identified seven such sites, which MEME and another phylogeny-based method, FEL [57], did not. Mutations in such sites are good candidates for changes that confer a profound selective advantage, due to immune selection, viral fitness, or both. Once they are introduced, lineages that survive to later time-points all carry the mutation.

In addition to site 411 in the CTL epitope, MEME identified 27 more sites with *p*-values below 0.1 and *q*-values above 0.2 (Figure S2), which LASSIE did not find. Most of these mutations were transient, rare, or not obviously related to the epitopes we know were targeted in CH505. For example, many of these sites occurred in gp41 (Figure S2).

#### 2.1.5. Threshold Considerations

To consider systematically what sites might be missed by the 80% peak TF-loss criterion we used here, we explored the locations of mutated sites that did not attain 80% TF loss at any time-point in the study period. The 365 sites that varied were dispersed over the entire protein, as expected. In particular, sites that had only one mutation among all available sequences are scattered throughout the structure. By requiring multiple mutations among all 385 full-length Env sequences, regional patterns appeared in the spatial distribution of mutations (Figure S3). Positions with as few as three or four mutations began to show a clear focus towards the immunologically targeted regions. This suggests that the relatively high threshold of 80% TF loss at any one time-point excludes from consideration some mutations that occur in immune-targeted regions, which may have phenotypic consequences for immunological sensitivity. Several more localized clusters of sites were apparent, which contained variable positions but did not attain the within-time-point 80% TF loss cutoff criterion (Figure S3). Such structural clustering suggests presence of other selected regions. However, these sites were not under the same high degree of selective pressure as sites in which the TF form was depleted. These sites may be targets for transient or less potent antibodies, or antibodies that are just beginning to impose selective pressure at the end of the study period. They might otherwise result from CTL escape, tolerance of neutral variation, or other mechanisms of molecular change not directly related to immune selection. LASSIE allows investigators to target the most highly selected sites for further study, and adjust the threshold as practical for reagent design.

#### 2.1.6. Concatamers of Selected Sites

A concise representation of selected sites strings the sites together to form concatamers of 35 amino acids. The order of sites in concatamers can, but need not, follow the primary Env sequence; here we ordered them by when non-TF mutations first emerged. Modified sequence logos [58,59], in which symbol height indicates frequency in a sample and the TF form is optionally left blank, clearly show this progression over time (Figure 5). This depiction helps to identify the timing and onset of various forms of immune response. The left-most column corresponds to the Loop D mutation in position 279, previously described [15] to emerge by day 28 in response to CD4 binding-site antibodies that contact this region of the viral envelope. Position 281, another Loop D mutation, is the third site to emerge in this ordering and appears as early as day 57. Positions 332 and 334 also change conspicuously early, emerging at day 141 and prevailing by day 157. These sites correspond to the well-studied V3 glycan shift [16,41], which is associated with neutralization escape from antibodies with V3-glycan specificities [27,60,61].

Multiple sites inserted together in the V1 hypervariable loop (144g, h, and f) also appear at day 141 and prevail by day 157. The diversity of insertions in the V1 hypervariable loop is complicated and detailed in Table S1. The representation as logos provides a clear and simple indication of the onset of evolutionary activity in this region.

Similarly, sites 462-465 in the V5 hypervariable loop appear in this scheme at day 211. Table S2 details other V5 hypervariable loop polymorphisms. Using the concatamer representation with sequences from other subjects whose immune specificities have not yet been comprehensively mapped suggests a promising opportunity to suggest which samples may contain the earliest evidence of immune activity.

The top row (Figure 5a) summarizes variant frequencies per site from Envs sequenced over the first three years of infection in this individual. Below that (Figure 5b), rows were stratified to summarize frequency in each sample, first for the TF virus (day 0), then for 14 plasma samples (day 28–day 1121, *i.e.,* week 4–week 160, post-infection). Electrostatic charges of amino-acid side chains, depicted by symbol colors (cf. Figure 2), changed polarity in 25% of the gp120 sites (279, 144h, 463e, 460, 347, 356, 275, 147) but not in gp41 sites. Gain or loss of potentially glycosylated asparagines (O) appeared in 13 of the 32 (41%) gp120 sites, but none of the gp41 sites. The swarm of sequences selected by the next stage of analysis (Section 2.2) was also depicted in this manner (Figure 5c).

Comparing timing of TF loss in the CH505 virus population with neutralization titers assayed longitudinally from contemporaneous plasmas (Figure S4) suggests that neutralization breadth follows Env diversification in selected sites. Autologous neutralization is evident at week 14, and heterologous neutralization breadth continues to increase thereafter. TF loss at selected sites starts to emerge at week 4 and net TF loss at all selected sites combined continues to increase until the end of the time-period studied.

### 2.2. Swarm Selection

#### 2.2.1. Representative Variants among Selected Sites

The algorithm identified 54 Envs that covered variant diversity at the 35 sites selected by TF loss. Table S3 summarizes these as concatamers. The LASSIE algorithm selection criteria had at least two clear consequences. First, the gradual accumulation of mutations found in early infection was deliberately mimicked using this strategy. Second, the appearance of each new mutation of interest is, by design, relatively isolated from other accumulating mutations emerging in the within-host virus population. Therefore, to the extent possible with the given sampling, each mutation in each selected site was expressed in a context as close as possible to the form of the Env in which it appeared when it first began to emerge in the viral population at a level high enough to be sampled. Thus, if a particular mutation conferred a phenotypic change in either antigenicity or neutralization susceptibility of an isolate, then that change would be included for study in its natural context. Selected mutations that appear early, but are retained at later time-points, are resampled together with later variants (Table S3).

Swarm variant frequencies (Figure 5c) resembled variant frequencies sampled in the virus population (Figure 5a), with additions of under-represented mutations at selected sites, which were less readily apparent in the larger population. Mutations seen only once among all of the sequences obtained were not required for inclusion, but all mutations in selected sites seen in two or more of all the sequences were represented by the 54 selected Envs. Mutations that occurred only once were not considered, as they are more likely to represent random mutations or possible sequencing error than recurring mutations. Increasing this setting to include each mutation only if it occurs more often will decrease the number of Envs selected.

#### 2.2.2. LASSIE Compared with Randomly Selected Sequences

**Figure 6:**
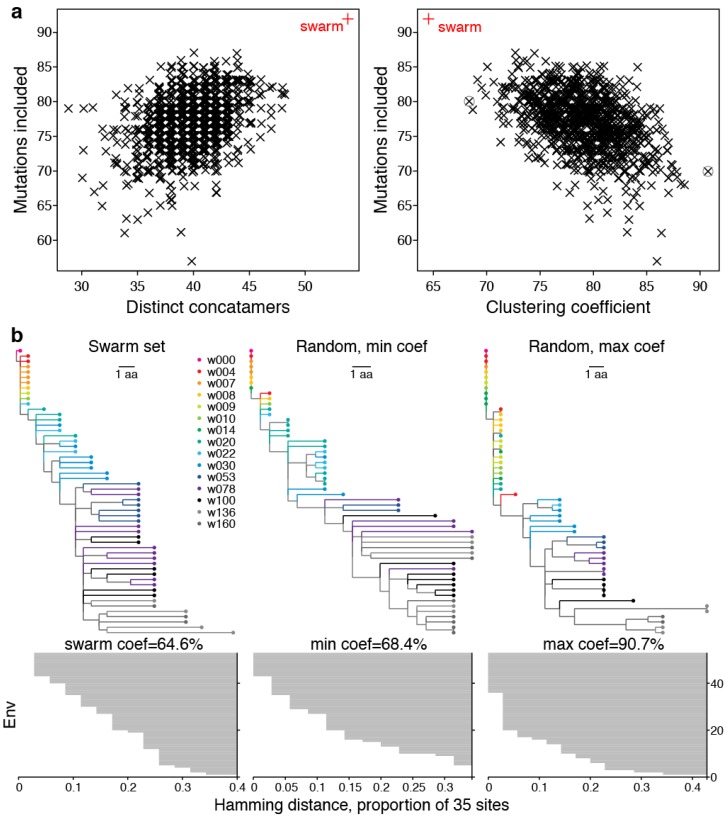
The selected swarm set is distinct from randomly selected sets. (**a**) Number of distinct concatamers, mutations included, and clustering coefficients from dendrograms of concatamer distances differ for the selected swarm of 54 Envs (red) and the null distribution from 1000 sets of 54 Envs, randomly selected without replacement from the non-redundant set of 260 viable full-length Envs, with the TF form always included. Values have jitter added for less overplotting; (**b**) Clustering coefficient quantifies sequence differences as the average normalized distance at which each sequence is merged into a cluster (horizontal grey bars in bottom row), compared for the selected swarm set two extreme randomly sampled sets (min and max, circled points in **a**, right).

We performed a resampling experiment to evaluate the swarm-selection algorithm against a null distribution, which might be sampled naively by less informed methods. The null distribution was sampled randomly from full-length Envs that had been normalized to eliminate multiple copies of the same Env sequence. Removing duplicates and excluding Envs with premature stop or incomplete codons gave 260 distinct Envs, from which we repeatedly resampled the same number of sequences as in the swarm set (54 Envs) without replacement. Figure 6 compares the null distribution from resampled results with the algorithmically chosen swarm. By design, in our set of 54 selected Envs, no concatamers were duplicated, *i.e.,* each Env carried a distinct combination of amino acids in the 35 positions of interest, and all recurrent mutations in selected sites were represented. Because the sites represented progressive adaptation of the virus in CH505, we expect each concatamer to have distinct antigenic and/or phenotypic properties, including sensitivity to the coevolving antibody response, which could be identified by assaying each variant against longitudinally obtained plasmas or mAbs isolated to represent a developing clonal lineage (Section 2.2.4). In contrast, among the randomly chosen sets of 54 Envs, redundant concatamers of selected sites were common. Resampling 1,000 replicates gave a median of 40 distinct concatamers with 95% CI from 34 to 45 (Figure 6a).

We also compared how many of the non-TF mutations tabulated in the first pass of the algorithm through all 385 sequences were covered. The antigen swarm set selected using LASSIE was designed to cover all 92 distinct mutations that arose in the 35 selected sites. As expected, random sampling of Envs gave consistently lower coverage of the mutations of interest (median 77; 95% CI: 69 to 84) than the 92 mutations that were included by the swarm-selection algorithm (Figure 6a). This indicates that random sets of the same size do not capture all of the mutations we consider to have the most potential relevance to immune selection in general, and antibody sensitivity in particular.

Further, we computed hierarchical dendrograms from Hamming distance matrices for swarm and random sets, and summarized the outcomes as clustering coefficients. The results shown here were obtained using the single-linkage method, which is related to the minimum spanning tree [62]. The strength of the clustering can be measured as a dimensionless number between zero and one called the agglomerative coefficient [63]. It is the mean normalized distance at which each sequence clusters with others, and characterizes how well the data are clustered together. To provide an intuition for how this coefficient works, Figure 6 also shows the dendrogram from the swarm set and compares it with the resampled sets that gave lowest (“min”) and highest (“max”) coefficients. The LASSIE selected Envs had a lower clustering coefficient (65%) than sets of randomly selected sequences, which had a median of 79% and 95% CI: 72%–80% (Figure 6). The lower clustering coefficient indicates less hierarchical grouping structure, *i.e.,* a more uniform distribution over the available sequence space or lower overall relatedness among subsets of concatamers from the selected Envs, than exhibited by the random sequence sets [63].

These metrics compared sequence sets from the swarm-selection algorithm with null distributions that were obtained by random selection. Because the three metrics are only loosely correlated, they measure different aspects of selected sets of sequences. To our knowledge, this is the first attempt to establish criteria to quantify how well any subset of sequences from a larger related set represents diversity (number of distinct concatamers), polymorphisms (number of recurrent mutations included), and progressive divergence (clustering coefficient) of the larger set. We suggest these objective criteria may be useful to choose representative sets of sequences, regardless of how they may be chosen; the smallest set of sequences that maximizes these three criteria, given an ordered list of selected sites, would then be considered the optimal sequence subset when choosing representative reagents.

#### 2.2.3. Phylogenetic Context

**Figure 7:**
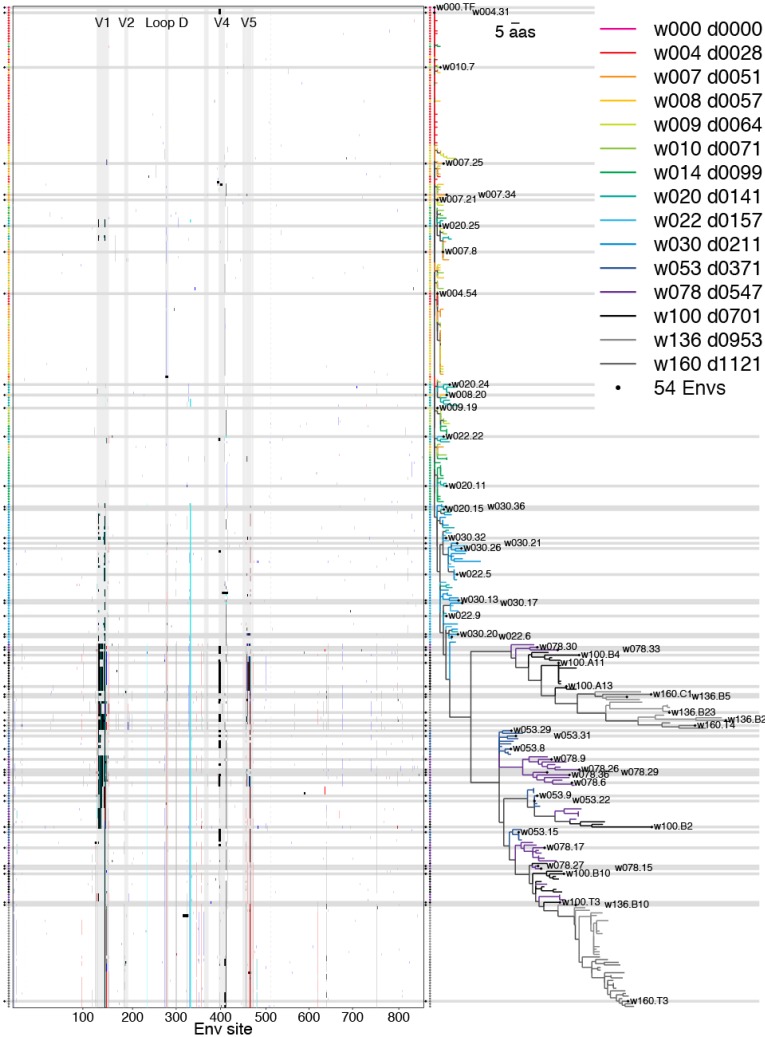
Env variants in phylogenetic context. A pixel plot is paired with the maximum-likelihood phylogeny, such that each row depicts one of 385 Envs sequenced by limiting-dilution PCR. The top row corresponds to the TF virus. In the pixel plot (left), sites that match the TF are blank and mutations are shaded indicate gain of negatively (red) or positively charged amino acids (blue), addition of an N-linked glycosylation motif (cyan), indels (black), or other mutations (grey). The colored vertical stripes that emerge with time correspond roughly to TF loss. Env landmarks appear as vertical bands throughout the pixel plot (light grey), and dashed line delineates the boundary between gp120 and gp41. Tree branches and symbols are color-coded to indicate sample time-point, and the 54 selected Envs are marked by a black circle and horizontal bar. HXB2 numbering is used here and throughout, beginning with the Env signal peptide start codon in column one and ending with the stop codon at position 857. This visualization was produced by the pixelgram package; see Section 4.4 for availability.

A consideration of the phylogenetic context of Envs included in the swarm set shows the persistence of selected sites against a scattered background of ephemeral mutations (Figure 7). Each row represents an Env and each column in the pixel plot corresponds to a codon in the Env alignment. The large blank region with zero-length branches towards the top of the figure correspond to the TF virus expanding clonally before the onset of immune selection. Each of the vertical stripes that appears in the pixel plot shown on the left-hand side of Figure 7 represents a site that exhibits a high prevalence of mutations relative to the TF virus. Viewing these sites in the full-length protein context helps to identify mutations that occur together. The swarm-selection algorithm chooses a sequence to represent each recurrent mutation in each of these stripes, favoring the top of each stripe, where the off-stripe mutational background most resembles the TF virus.

Selected Envs were widely distributed over the phylogeny. The earliest selected Envs (weeks 4–10) tended to carry single mutations, which are often carried forward and represented again by Envs at later time-points. Some later Envs represented large clades sampled only in one or two time-points, such as the sequence w160.T3 (KC247482), which appears almost at the bottom of the tree (Figure 7).

Two sequences from CH505 (KM284795 and KC247403) with aberrant in-frame insertions, disrupted the x-axis linear scaling, as in Figure 1. These two sequences were deleted from Figure 7 but retained in the alignment.

#### 2.2.4. Antigenic Diversity

We opted to work with data from subject CH505 for initial LASSIE development and testing not only because a large set of longitudinal sequences was already published and available, but also because roughly one-fourth of the Env sequences from CH505 had been hand-selected and previously expressed as pseudovirus and proteins, then subjected to immunological evaluations [15,16]. These available data provided an opportunity to explore the antigenic diversity and neutralization sensitivity represented by a large subset of the antigenic swarm that LASSIE selected. Of note, the sequences we had originally used to analyze the CH505 immune response had been chosen manually from the phylogenetic tree, a practice typical in this field. Combined uncertainty about how well the manual selection covered all immunologically relevant mutations in CH505, how effectively it recapitulated the gradually emerging resistance to evolving antibody clonal lineages, and a desire to minimize the expense of building very large subject-specific Env reagent sets in future studies, all motivated development of LASSIE. LASSIE provided a principled solution for the first two of these biological problems, coverage and mimicking *in vivo* antigen evolution, while defining a minimal set of Envs to achieve these ends, and minimize experimental costs.

**Figure 8:**
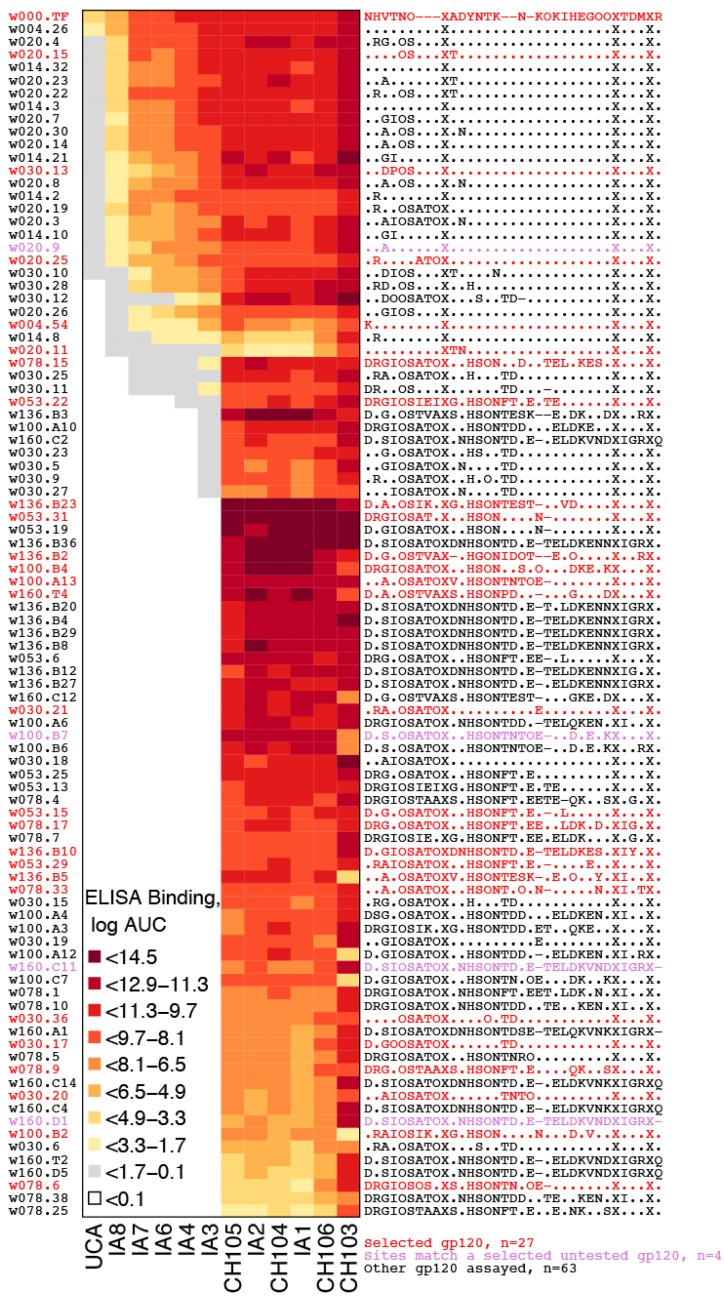
Selected Envs represent diverse binding phenotypes. Among the swarm of 54 Envs selected, 27 were synthesized as gp120s for ELISA binding assays (red text). Another four of the antigens tested contained selected sites that matched with those in selected Envs (purple text). Binding data are shown as colors to indicate log-transformed area under the curve (AUC) from dilution series, which summarized experimental results better than EC^50^s. Both assays tested Env constructs against monoclonal antibodies of the CH103 lineage, from mAb isolates (e.g., CH103) to the unmutated ancestor (UCA) via intermediate ancestors IA1–IA8 [15]. Blank entries indicate no binding was detected. Selected Env sites correspond to concatamers in Table S3. An “X” appears for gp41 sites, which were not in the gp120 antigens tested. Data are listed in Table S4.

ELISA binding assays with mAbs from the CH505-derived CH103 CD4 binding site bnAb B cell lineage were available for 94 gp120s; a subset of 27 LASSIE-selected Envs was included in this set (Figure 8). Binding assay results confirmed that selected viruses exhibited diverse antibody sensitivities, which increased with maturation of the bnAb lineage and generally followed the progression of mutations away from the TF virus (Figure 8).

In a similar manner for neutralization sensitivity, 26 LASSIE-selected Envs were among 121 Env-pseudotyped viruses tested for neutralization sensitivity by CH103 lineage mAbs [16] (Figure S5). Selected Envs represented the range of sensitivities among viruses tested, reflecting the diversity of variants that developed in response to sustained selection for neutralization escape.

In Figure S6, neutralization titers from all previously hand-selected viruses clearly show the development of neutralization breadth in phylogenetic context. Envs at the top of the tree are broadly susceptible to many antibodies in the CH103 lineage. Envs that evolved later appear lower in the tree. Neutralization breadth was acquired later in bnAb ontogeny, which is clear as a gradient of increasing potency from the unmutated ancestor (left) to the mature CH103 bnAb (right). By selecting Envs that represent genetic diversity sampled during bnAb development, the method selects Envs that represent relevant antigenicity over time.

### 2.3. Swarm Size Adjustments

We have used the LASSIE approach to analyze samples from other individuals with larger data sets available. To reduce the number of Envs for inclusion in reagent design below 100 from over 1000 initially sampled sequences (as compared to 385 sequences from CH505) required that we increase the TF loss cutoff above 90%. Selecting an antigenic swarm of fewer than 100 sequences also required increasing the frequency of rare amino acids to be represented in the swarm. Both of these adjustments focused outcomes on sites that ranked highest in terms of selective pressure (TF loss threshold) and the mutations within these sites that are most successful under host immune pressure (minimum variant count).

Another way to reduce the number of sites and sequences that result from LASSIE is to exclude sites that were insertions relative to the TF sequence, which occurs most frequently in the hypervariable loops. Due to the evolutionary processes that yield length polymorphisms, rather than point mutations, and the resulting difficulty of consistently aligning homologous sites in these regions, we naturally found disproportionately high diversity of V1, V2, V4, and V5 sites among selected Envs. Resources to produce reagents that capture each recurring point mutation in the hypervariable loops might be better allocated elsewhere. Excluding such sites from the list of selected sites used to choose sequences reduced the number of sequences, while still representing diversity of envelope regions with well-defined structure. However, because these length polymorphisms in variable regions are known to be involved with immune escape (e.g., [53]), representation of the most common forms found in the hypervariable regions should be still included in swarm sets. When exploring the option of excluding TF insertions from the selection criteria, we found that different forms of hypervariable loops were indeed still included in the antigenic swarm, but their representation was of course limited to evolutionary contexts in which they occurred together with sites that have high TF loss. When considering this alternative, one should ensure that the common forms of hypervariable-region variants, such as we have listed in Tables S1 and S2 as the most common forms observed, are included among the antigenic swarm identified by LASSIE. If not, they might be added as specific additional sites or sequences to the reagent set (see Section 4.2 and Section 4.3).

### 2.4. Chronic Infection

These methods were developed initially to select sequences from longitudinal studies beginning early in infection, where the TF virus is reliably inferred, and the progression of escape mutations is readily apparent. This is obviously not true for chronic infection. Still, it is often necessary to select a subset of sequences that represent diversity in serial samples taken during chronic infection. To evaluate the algorithm’s ability to select an antigenic swarm from a chronic infection, we applied it to sequences from a study participant enrolled during chronic infection, designated CH0457 [41]. We analyzed 205 plasma SGA Envs from 10 sample time-points (median was 20 sequences per time-point; the distribution ranged from 12 to 35). In the chronic enrollment sample, the first available, five of twenty Envs exactly matched the within-time-point consensus. We used one of these as the reference to compute variant frequencies. No variation was detected in 582 of 888 aligned sites, and an 85% cutoff identified 35 sites that were candidates for strong positive selection (Figure S7). Nine of the 35 selected sites are located in gp41.

With singleton variants excluded, the algorithm selected a swarm of 44 Envs (Figure S8). The progressive accumulation of mutations among concatamers of selected sites is less clear in this chronically infected subject than in acute infection (cf. Figure 5b). Furthermore, sites that appear to be under selection in the interval sampled are not clearly associated with two epitope regions, as was the case of CH505, where there was a strong imprint of CD4bs and V3 antibodies selection, and indeed antibodies with these specificities were isolated from the subject. In the case of CH0457, most of the selected sites were not identified as relevant to known antibodies, although two sites were in the MPER region of gp41 (HXB2 positions 667 and 671) and two sites were predicted signatures of the 2F5 MPER antibody (positions 640 and 351). In addition, one site was in contact with some CD4bs antibodies: a changing glycosylation pattern at 461, which contacts CD4 and the CD4bs bnAbs VRC01 and NIH45-46. Two of the selected sites (651 and 640) have been noted to be CD4bs antibody signatures [47]. A potent CD4bs bnAb CH27 was isolated from subject CH0457, but the virus isolated from CH0457 plasma had escaped from this antibody by the time of enrollment [41]. However, archived provirus from CH0457 cell-associated DNA remained sensitive to neutralization by bnAb CH27 [41]. CH13, a weaker CD4bs nAb capable of neutralizing only heterologous Tier 1 viruses, was isolated, and may have been exerting selective pressure in the last weeks sampled [41].

The phylogeny indicated a persistent, divergent secondary clade, represented by 24 of 205 plasma Envs (Figure S9). This clade was not introduced by misalignment nor by simple recombination, and was also represented by cellular provirus sequences [41]. Though the divergent clade was undetected among sequences from the enrollment sample, it was represented by 14 of the 44 Envs selected (Figure S9). Thus, the algorithm can be applied to both acute and chronic Env sequential sequence analysis and swarm design.

## 3. Discussion

Vertical stripes of mutations in an aligned set of serially sampled sequences, as shown in Figure 1 and Figure 7, indicate sites where the transmitted-founder amino acids are lost over time, a useful criterion to identify candidate selected sites. We have presented a systematic approach to identify these sites, track their dynamics graphically, and then identify protein sequences that capture mutations in the selected sites as they first emerge in the quasispecies. The task of selecting representative variants from a larger set for follow-up studies from longitudinal samples can be complex when choosing from hundreds or thousands of sequences. LASSIE divides the task into two main parts, automatically identifies and tracks selected sites within a subject, and identifies sequences that represent antigenic diversification in that subject.

### 3.1. Site Selection

First, we use transmitted-founder loss in a longitudinal study as a simple way to identify sites under positive selection pressure. Despite the existence of a variety of methods to test for positive selection by comparing rates of synonymous versus non-synonymous substitution, their utility to identify sites under positive selection in the limited diversity context of within-subject viral evolution is limited by statistical power [56,64]. For example, the strong selective pressure may be exhibited by a single change in a phylogenic reconstruction, as illustrated in Figure S1. This could happen with a population bottleneck, or an advantageous mutation or mutations that are strongly favored being carried forward through recombination. Also, allele frequencies can be biased by removing identical sequences in order to obtain dichotomously branching trees. Identical sequences are unlikely to occur among samples from different hosts, but commonplace when sampling from acute through early infection within a host [5,15,16,65,66].

In contrast, loss of the TF form at any time-point is a simple and inclusive measure. In CH505, sites selected by this criterion were focused in regions highly relevant to the known adaptive immune responses previously identified in the subject [15]. This suggested that in future studies, structural localization of selected sites could be used to raise hypotheses about specificities of bnAbs in plasma. Furthermore, the timing of TF loss identifies these important mutational events and could help determine when antibodies are exerting the most selective pressure, indicative of which samples in a longitudinal series are likely to yield antibodies with particular specificities, and when the earliest members of a clonal lineage that target a particular epitope first appear. Such information could help efforts to isolate monoclonal antibodies in subjects with potent nAbs, by focusing on antibody specificities that recognize the epitopes under selection, and by identifying which samples might best be used to isolate new bnAbs from subjects sampled over several years of follow-up.

Admittedly, the TF loss criterion alone cannot distinguish between sites that represent a random sweep, in which chance establishes a mutation in a lineage, and selected sites. This is why we refer to the set of sites LASSIE identifies as *candidates* for sites under positive selection. For example, LASSIE could identify a neutral mutation genetically linked to another mutation under positive selection. We aim to be inclusive and evaluate immunological phenotypes of such patterns in our swarm sets, to ascertain which mutations are indeed escape mutations. In this way, LASSIE is designed to minimize type II errors, even with a small cost of increased type I error. Parameter settings can be chosen based on both this trade-off of sensitivity with selectivity and available resources. Also, even if a mutation were neutral when it first arose, and subsequently maintained in the population, it may represent a pre-adaptive state that allows novel escape mutations to accrue that depend on the presence of the initially neutral mutation. LASSIE “swarms” bring each new mutation into the swarm in the Env genetic context in which it was first sampled, in the simplest form possible, relative to the evolving quasispecies. Thus, subsequent immunological experiments can identify immune-sensitive phenotypic profiles between Envs with minimal amino-acid changes between them. This helps to isolate the candidate sites under pressure from immune selection, to the extent possible in the context of appropriate Envs.

### 3.2. Sequence Selection

Second, we provide a rational, objective method to guide the selection of Env sets for experimental study from large sequence sets sampled over time. LASSIE can select sets of sequences that represent gradual antigenic diversification induced during bnAb development, ensuring that all variants in sites identified by TF loss are represented in an Env reagent set, while minimizing redundancy, and selecting only as many variants as are necessary to represent diversity in sites selected by TF loss. For a set of aligned sequences sampled from an acute, single-founder infection, the algorithm starts with sequences most like the form that established the infection, and gradually increases diversity in a manner that parallels natural infection. Though a smaller set of sequences could be defined that minimally covers all variants at selected sites, the gradual accumulation of mutations would not be captured by such minimal set, and more subtle transitions may be critical for selection of bnAb breadth, *i.e.,* evolving antibodies may adapt more readily to serial introductions of single mutations in an epitope, rather than to those same changes introduced simultaneously.

We used LASSIE to identify selected sites and representative sequence subsets in longitudinal samples from one acutely infected subject and one subject sampled only during chronic infection. We analyzed SGA sequences, which provide intact *env* gp160 genes with no recombination artifacts and minimal error [5,14,21,65,66,67]. While such data provide ideal conditions, the approach could also be used in other longitudinal study designs and sequencing strategies (e.g., [68]).

In related research, sequence selection has been represented as a set-coverage problem [69], to identify networks of covarying sites in a population-level alignment, which represents a particular clade [70], rather than a serially sampled, within-subject alignment as in this study. A limitation of our approach, which we intend to address in the future, is that sites are treated independently, while covariation between sites may influence variant suitability and TF loss. Considering covariation may potentially facilitate identification of smaller representative swarm sets, and may better accommodate rampant recombination between divergent lineages within a host.

By progressively adopting mutations in the context of variant sequences where they first arise in our sequence sets, our swarm sets—by definition—allow the study of mutations in the context of the natural combinations of mutations as they occurred *in vivo*. This strategy could complement, or in some instances provide sufficient information to replace, traditionally used site-specific mutagenesis, which necessarily studies mutations in isolation. As mentioned above, a mutation observed in a later time-point and introduced into the TF, for example, may not be viable or may not have the same phenotypic consequences as it does in the background of the Env in which it arose, so the ability to study related natural variants isolated serially may ultimately be more informative.

A working hypothesis to explain the observation that bnAbs tend to arise late infection, after antigenic diversification has arisen in the subject, is that serial immune escape *in vivo* drives antibody lineages to adapt to the emerging viral variants, and eventually enables recognition of diverse forms of the targeted epitope from the circulating population [16,18]. Exposure of a neutralizing antibody lineage during affinity maturation to increasing antigen diversity could result in selection of antibodies with increased breadth [15,18,31,37,38]. Mimicking *in vivo* diversification has therefore been proposed as a possible vaccination strategy for bnAb induction [15,18,71,72,73]. Related work has suggested that Env variants sampled during development of heterologous neutralization breadth could be administered as immunogens [74,75,76].

With recent technological advances, it is becoming feasible to test vaccine designs that not only include five to 10 antigens, but potentially 50–100 antigens, administered as DNA either in series or in combination [77,78]. Because LASSIE uses an efficient algorithm to identify candidate sets of antigens with progressively increasing diversity at important sites in polymorphic viral proteins, it could be used to help with the design of such antigen-swarm vaccines. This method could be applied to other large, longitudinally sampled sets of sequences, such as from hepatitis C virus [79,80,81,82,83]. Use of this method to analyze antibody sequences, which complement the evolving viral sequences, could identify selected sites and select a representative subset of sequences from antibody clonal lineages.

## 4. Materials and Methods

### 4.1. Overview

LASSIE analyzes a protein sequence alignment in two phases. The first phase identifies protein sites most likely to be under positive (diversifying) selection, by considering the extent to which the TF amino acid state is “lost,” *i.e.,* replaced by mutations or deletions, at any one time-point during longitudinal sampling. This yields a list of sites of interest, from which we tabulate and track amino acid mutations that appear over time. TF loss is a simple and useful strategy to identify candidate sites undergoing positive selection in a scenario such as HIV evolution *in vivo* [68]. Profound immunological pressure can result in a selective sweep, where a mutation in a site is fixed and persists after its initial introduction, and such a mutational event may occur only once in a reconstructed phylogenetic history. Such sites may be critically important to the evolving immunological phenotype of the virus, and would be good candidates for attention in experimental work. However, they would be undetected in commonly used statistical methods to detect positively selected sites. This is because the common methods require recurrence of mutational patterns in a tree for statistical validation, to identify ratios of non-synonymous to synonymous mutation rates significantly greater than one [56,84,85].

In contrast, our goal is to identify inclusively the sites that are *candidates* for being under positive selection pressure, as quantified by the extent of transmitted-founder loss [68]. To motivate use of the TF loss criterion, we performed a detailed comparison using both MEME [56] and LASSIE with longitudinal sequences from CH505, described in Results. LASSIE also provides plotting and visualization functions to review variation in selected sites over time. Comparing the list of selected sites with previously described antibody contacts from the growing set of anti-HIV bnAbs can facilitate the search to characterize immune responses that drive sequence diversification in newly obtained samples. The second phase of analysis uses the list of selected sites to choose sequences that represent the natural accrual of mutational variants that occur more than once among selected sites. The two phases of analysis, and parameters that influenced the number of sites and sequences thereby obtained, are detailed below.

An important feature of the single-genome amplification (SGA) sequences we analyzed is that they were obtained by limiting-dilution PCR, which provides genetic linkage across all of the *env* gp160, without *in vitro* recombination artifacts, and with limited nucleotide substitution errors during cDNA synthesis [66,67]. Unlike Sanger sequencing from bulk PCR or large numbers of fragmentary high-throughput reads from unlinked templates, SGA sequences can provide high-quality sequence data spanning intact genes, ideally suited to understand how viruses adapt to host immune responses over time [5,14,21,65,66,67].

### 4.2. Site Selection

We analyzed *env* cDNA amplicon sequences from plasma viral RNA by single-genome amplification (SGA), also known as limiting-dilution PCR [67], sampled longitudinally, beginning early (4 weeks) after infection, with three years of clinical follow-up. The sequencing effort was intended to obtain about 20 sequences (median of 25, range 18–53) from each of 14 samples. This yielded 385 full-length sequences in total. SGA sequencing from homogeneous infections commonly yields multiple identical sequences, all of which we kept. (Because they produce “rakes” of identical sequences on phylogenetic trees, monotypic sequences contain information about evolution and allele frequencies in the population sampled, but are incompatible with methods that require phylogenies with strictly dichotomous branching. We revisit this issue in the Discussion.) A naming convention ensured consistency and enabled parsing of sample time-point labels from sequence names. By default, time-point labels are assumed to be in the first dot-delimited field of each sequence name, though any character could separate fields and time-point labels need not be in the first position. For homogeneous infections sampled before the onset of immune selection, such as subject CH505, a simple Poisson model of random sequence evolution estimates days post-infection using sequences from the earliest sample [5,6]. To plot variant frequencies as a function over time, we added the estimated number of days post-infection to the number of days between sample dates.

We added the HXB2 reference sequence to standardize site numbering, codon-aligned the sequences, translated them, and inferred a phylogeny. Subsequent analysis used the translated, aligned Env sequences that resulted. Accurate alignment of the hypervariable loops, which evolve by insertion and deletion, is difficult for automated algorithms. Because no algorithm aligns the HIV envelope perfectly, particularly when a translation is needed, we manually edited the preliminary alignment, which was based on an HIV-specific hidden Markov model [86,87], for consistent and compact loop regions. PhyML v3 inferred maximum-likelihood trees from translated amino-acid sequences with the HIVw (HIV-specific, within-host) substitution model [88,89,90]. The phylogeny was used to order sequences for visualization and provides an organizing principle for sequence evolution from the ancestral TF virus. Reordering the alignment to follow the phylogeny was used to review alignment accuracy and consistency among closely related sequences. To identify potential N-linked glycosylation (PNG) sites readily, we annotated PNG sites, replacing asparagine sites that match the Nx[ST] motif to become Ox[ST]. In the PNG motif, x indicates any amino acid except proline, and the third position is either serine or threonine. To identify selected sites, the gaps inserted to maintain an alignment are considered a character state, because HIV frequently evolves by insertion and deletion, in addition to point mutations. For each aligned site, we computed TF loss per time-point sequenced, identified the maximum, and compared this peak TF loss with a threshold. We adjusted the threshold and considered the resulting number of sites. This produced a list of amino-acid sites, which we considered as interesting evolutionary “hot spots”, to be represented by a swarm of Envs for experimental design.

We identified selected protein sites by transmitted-founder (TF) loss, defined as the proportion of sequences sampled per time-point that mutated away from the ancestral TF state. This is an efficient and inclusive way to identify rapidly evolving sites [68]. Because immune pressures can be transient, a variant may be relevant in only a particular phase of antibody lineage development. Thus, by considering TF loss per time-point, we seek to identify mutations that may be transiently important for resistance profiles of developing B-cell lineages. Here we considered no other information than TF loss per time-point, though other information could be used, so an option to specify additional sites for inclusion with the automatically selected sites is available. For example, signature sites associated with neutralization assay outcomes, antibody contact residues from structural data, key glycosylation sites associated with common bnAb epitopes, and additional sites identified using other methods (e.g., [56,84,85]) can be included by listing them for subsequent evaluation, even when these sites do not meet the minimum TF loss criterion. Similarly, if excessive diversity in hypervariable loops needlessly increases the number of sequences selected, such sites can be excluded simply by listing them.

Calculations of cumulative TF loss weighed the TF loss for two consecutive samples by the amount of time elapsed between when they were drawn. Use of cumulative TF loss distinguished between sites that reverted to the TF form and sites that quickly mutated away from the TF and never reverted, and between sites that changed at different rates. For an informative representation of the accumulation of mutations among selected sites during site selection, we sorted sites by combining two criteria: time to initial TF loss and cumulative TF loss. The ordered list of selected sites identified automatically by LASSIE, or added specifically by choice, forms a “concatamer” by stringing together the listed sites extracted from a full-length sequence.

For identifying sites that are subject to selection, and determining when mutations in these sites begin to accrue, LASSIE analysis could pause here.

### 4.3. Sequence Selection

After having used the TF-loss criterion to select sites from the alignment, we identified a set of Envs to represent the mutations that occur at these sites. Choosing 50 representatives from 385 candidates gives over 10^63^ distinct alternatives. We know of no established optimality criterion to choose representative variants from a larger set of aligned sequences. The second phase of analysis provides a practical solution to the problem, which we consider as a working proxy for an optimal solution [91].

We designed and implemented a simple, efficient algorithm to select sequences that represent mutations at sites selected by TF loss. The approach is greedy, meaning it adds variants iteratively, rather than refining the entire set for potentially better solutions. Although such a greedy approach is unlikely to give the best possible overall solution, in situations where addition of variants leads monotonically to better solutions with diminishing returns, a greedy algorithm efficiently provides reasonably good approximations to the optimum solution [91], and can be refined to include other criteria as needed. The algorithm starts with the alignment used to select sites, and assumes that sample time-points can be identified from sequence names. A key feature of the approach is that, by considering the time of sampling, it starts with sequences most like the form that established the infection. It then progressively builds diversity in a manner that follows the natural course of infection. In this way, common mutations and mutations that eventually reach fixation are sampled repeatedly in varied genetic contexts.

**Figure 9:**
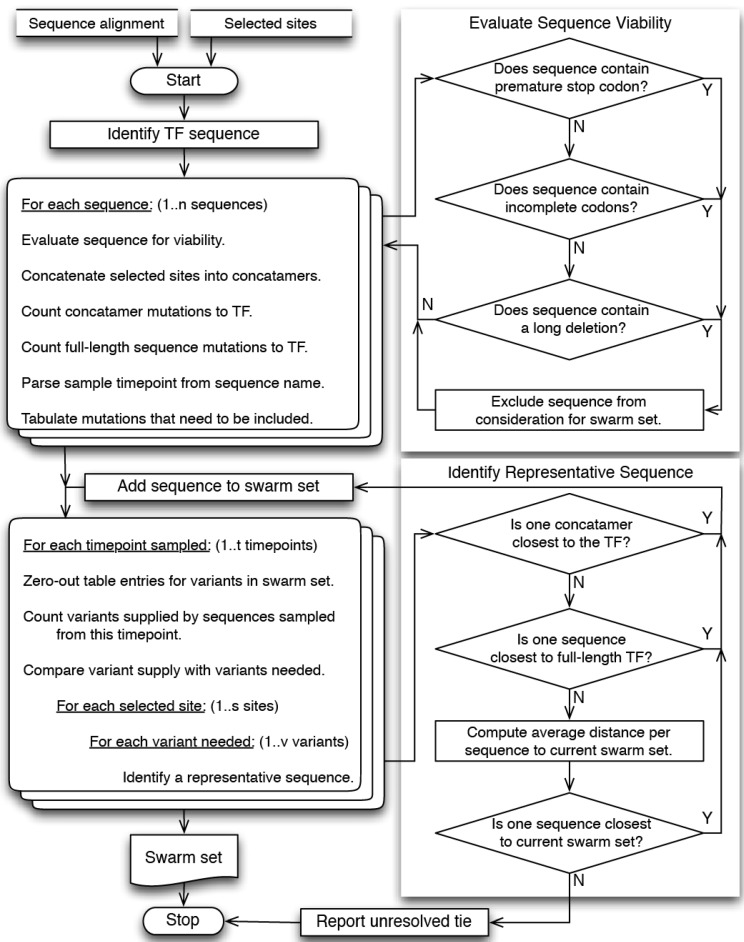
Swarm-selection algorithm. From a protein sequence alignment and list of selected sites, this approach identifies viable Envs and tabulates mutations in selected sites. The table initially defines which mutations will be represented by the swarm, and subsequently keeps track of which mutations remain to be included. Rare mutations, *i.e.*, mutations detected fewer times than the minimum variant count over the entire sampling period, are disregarded. Selection among multiple sequences that carry a mutation is resolved by minimizing a series of distance criteria, first to minimize Hamming distance (number of mutations, gaps included) to the TF form among selected sites, then distance to the full-length TF sequence, and finally to minimize average distance to sequences in the current swarm set. The selected Env is included in the swarm set, counts in the table of needed mutations are set to zero, to indicate the particular mutation is now covered in the swarm, and iteration continues. This produces a “swarm” of Envs, which represents diversity in selected sites as it developed within the subject, given sampling constraints. Stacked boxes signify iteration. Unresolved ties are reported, though we have not yet encountered them in several large experimental sequence sets we have tested; such an outcome would signal the need for an alternative distance metric or more selection criteria.

As outlined in Figure 9, the swarm-selection algorithm identifies sets of sequences that recapitulate viral evolution in key residues from a table of the amino acid mutations that occur at each site selected in the first analysis phase. Mutations that only ever appear once, or less than some other minimum number of times if specified, are disregarded. This parameter, the minimum variant count, is set to two by default. The resulting table lists all mutations to be included in the swarm set of Envs. Candidates for Env selection must be functionally viable, by lacking long deletions (as specified by the operator of the algorithm) and premature stop codons or incomplete codons, which typically result from frame-shift mutations.

Building a swarm set starts with the TF sequence. This can be identified when sampling from acute infection established by a single virion, which occurs among four out of five heterosexual infections [5]. If working with sequences that were first sampled during chronic infection, the natural sequence most similar to the consensus from the first available time-point is a good alternative. Sequences from the earliest time-point sampled are then considered, and scanned for the presence of sites with amino acids represented in the table of mutations. If a particular mutation of interest is present in one or more sequences from the first time-point, the choice among multiple Envs that carry a needed mutation is resolved by a series of criteria. The algorithm first tries to identify the sequence that uniquely minimizes the distance (number of mutations, including gaps) to the TF among selected sites. Then, in case of ties, a sequence is chosen that minimizes distance to the full-length TF. Finally, if ties remain, the sequence chosen minimizes the average distance to the current working set of sequences (Figure 9). An option exists to require that specific sequences be included, if desired. Such a sequence is added during iteration, when the time-point from which the sequence was sampled is being evaluated, rather than beforehand, to ensure inclusion of earlier (less divergent) sequences that carry mutations found on the specified sequence. After iterating over sample time-points, needed mutations, and selected sites, swarm selection is complete. Unresolved ties may exist among alternative sequences for some data. Such remaining ties would indicate a need for an alternative distance metric or additional selection criteria, though we have not yet encountered this outcome using Hamming distances from matching amino acid sequences and the three selection criteria described.

The algorithm is deterministic, which means it will always produce the same set of sequences from a given alignment, because it does not make random choices, and does not depend on the order in which sequences are provided in the input alignment. Overall, an advantage of this approach is that it selects no more sequences than are necessary to represent the mutational variants in selected sites, rather than some arbitrary number. By design, this greedy approach favors inclusion of early point mutations. This strategy produces larger sets of sequences than would result from favoring later, more divergent Envs that carry a greater number of needed mutations, but it better recapitulates the gradual increase of diversity in the evolving virus population. Among selected sites, each mutation observed more than once will ultimately be included in the antigenic swarm, generally multiple times if it remains common in subsequent time-points. The algorithm identifies the first appearance of mutations of interest in the least divergent sequence background possible, among available sequences sampled. It does this by progressively covering mutations that occurred in selected sites in the first time-point they appeared, and by representing them with the sequence most similar to the TF or, to resolve ties, the sequence most similar to those under consideration (lower-right quadrant in Figure 9).

The algorithm was made efficient through use of vector operations, and computes distance matrices only when they are needed to choose between otherwise ambiguous alternatives. Its duration of execution is expected to require no worse than a linear increase with the number of sequences and sample time-points in the input alignment. That is, doubling the number of input sequences or sample time-points should no more than double the run time. This was consistent with our experiences applying LASSIE to other large longitudinal data sets.

### 4.4. Availability

The methods to select sequences and sites and to plot the results graphically were written as an open-source (GPL v2) R package called lassie [92]. Development activities are underway to provide lassie as a web-enabled service via the LANL HIV database. The package includes aligned sequences from CH505 and a tutorial vignette. The methods used in this paper to plot variant frequencies are included with the lassie package. The package includes the CH505 alignment (GenBank accession numbers KC247375–KC247667 and KM284696–KM284799) as example data; *env* sequences from chronic infection in CH0457 are in GenBank (KT220796–KT221004).

To visualize variation among descendants sampled serially from within a host, we have paired phylogenetic trees (rooted on the TF virus, ladderized, then rendered as phylograms) together with pixel plots [93,94], which illustrate polymorphisms as either mutations or indels relative to the TF sequence. We find these to be informative representations for understanding evolution of the virus population in an acutely infected host, given the limited genetic diversity that occurs in early infections [15,41]. Renderings such as given above emphasize sites with evolutionary changes that produce the branching patterns in the tree, and enable detection of recombinant clades or evolutionary associations with phenotypic assays. The code we use to make such renderings was written as an R package called pixelgram [95], and uses ape to draw trees [96].

Others wishing to visualize their sequence data in a manner similar to the figures presented above are able and encouraged to do so with these tools. Simple installation instructions and tutorials that replicate the figures herein are provided at the two github addresses cited above. For best results, high-resolution vector graphics files are attained using the PDF driver in R.

### 4.5. Positively Selected Sites by MEME and FEL Analyses

We analyzed the CH505 *env* codon alignment using the DataMonkey server [97]. Nine sequences with premature stop codons were excluded. After removing these and the duplicated sequences, 333 sequences remained. Because this alignment was too large for GARD recombination analysis and used directly for MEME and FEL analyses [56,57], these results are limited by the caveat that recombination was not excluded. We used the GTR/REV model of base substitution [98,99], and considered all sites with *p*-values below 0.1.

### 4.6. Clinical Sample Assays

Procedures for binding and neutralization assays have been described elsewhere [15,16,41]. Clinical materials were obtained as a part of the CHAVI 001 observational study. All participants in that study gave their informed consent before they participated in the study, which was conducted in accordance with the Declaration of Helsinki. The Kilimanjaro Christian Medical Centre Research Ethics Committee, the Tanzania National Institute for Medical Research Ethics Coordinating Committee, and the Duke University Institutional Review Board approved studies involving human subjects from whom we obtained materials with results described herein.

## 5. Conclusions

In summary, we have developed computational methods to identify and track selected sites in longitudinal sequence data, and to use these selected sites to aid in down-selecting sequence sets for reagent design, or for testing the “antigenic swarm” vaccine concept. When applied to longitudinal HIV samples, a retrospective evaluation of viral sequences from the intensively studied subject CH505 showed that LASSIE provided meaningful results. High TF loss highlights mutations in selected sites that were indeed under immune selective pressure. An efficient algorithm builds a non-redundant collection of sequences tailored to characterize the phenotypic consequences of the mutations in those sites. LASSIE may be useful in many contexts, for reagent selection, to assist with bnAb isolation, potentially for vaccine design, as well studies of other viral infections, and studies of antibody evolution.

## Supplementary Files

Supplementary File 1
