# Supplementary material for: Longitudinal Antigenic Sequences and Sites from Intra-Host Evolution (LASSIE) Identifies Immune-Selected HIV Variants
Source: Viruses. 2015 Oct 21;7(10):5443–75. doi: 10.3390/v7102881 (PMC4632389; doi:10.3390/v7102881)
Supplement: Supplementary File 1 [file viruses-07-02881-s001.pdf]

viruses

MDPI

Supplementary Materials

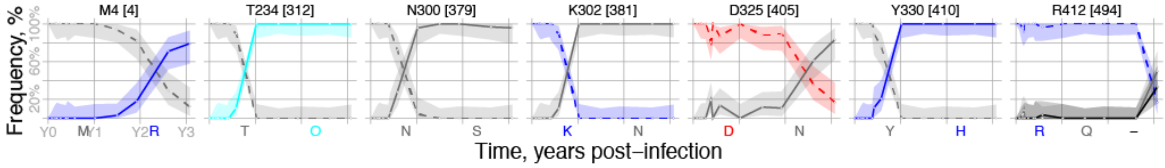

Figure S1. Sites with high TF loss not detected by MEME. As in Figure 2, the single TF amino acid (dashed lines with 100% initial frequency) yields to putative escape mutations (solid lines with 0% initial frequency) over the sampling period.The seven sites selected with over 80% peak TF loss that were not selected by MEME (q-values above 0.2 and p-values above 0.1) show abrupt transitions from the TF to a mutated amino acid, described as dynamic category *i* in Section 2.1.1 of the main text. For clarity, mutations that never attain 15% frequency in any sample are not shown. Shaded regions show 95% confidence intervals for variant frequencies, computed from the binomial probability distribution, given the number of sequences sampled per time-point.

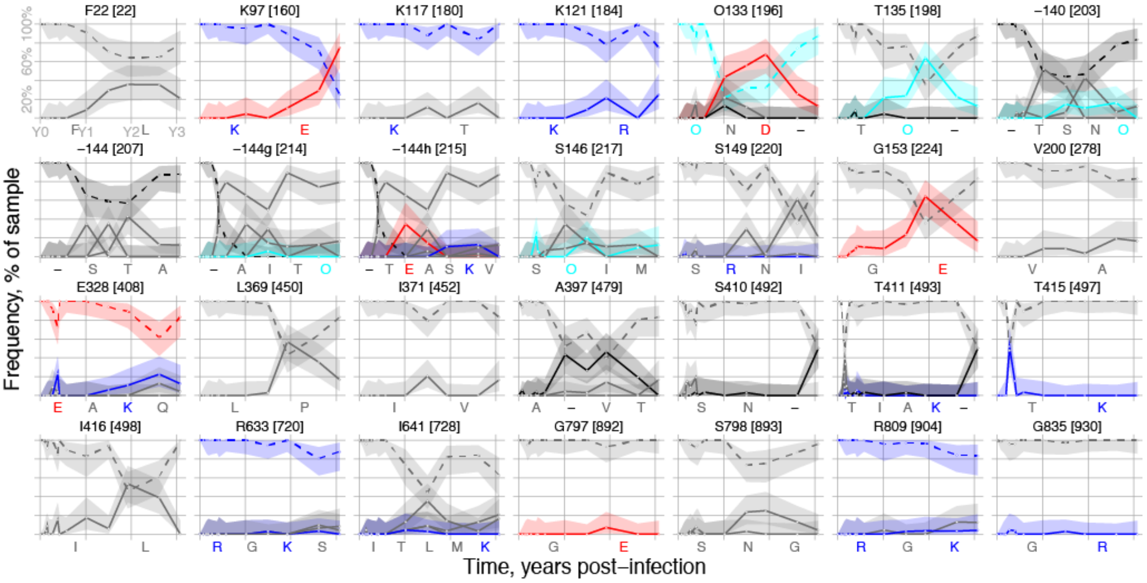

Figure S2. Sites without high TF loss detected with positive selection by MEME. Depiction of TF amino acid (dashed line) and putative escape mutations (solid lines) follows Figures 2 and S1. The 28 sites selected by MEME with q-values below 0.2 and p-values above 0.1, which were not selected by the 80% peak TF loss criterion, included dynamic categories *i*, *iii*, and *iv* described in Section 2.1.1 of the main text, and included at least four sites without dynamics. Shaded regions show 95% confidence intervals.

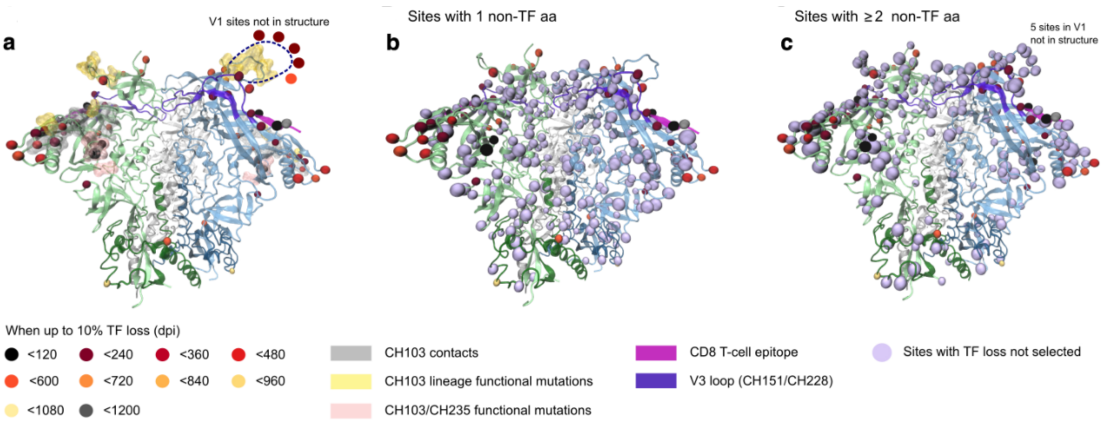

Figure S3. Locations of selected and non-selected Env sites in CH505. (**a**) Sites selected by high TF loss are depicted by beads, whose colors indicate when each site exceeded 10% TF loss, as listed in **Table 1**. For structural context, the immunologically relevant mutations and regions described in Figure 4 are also shown. V1 sites missing from the structure are illustrated schematically (top left). (**b**) Sites that mutated only once among 385 CH505 Env sequences (0.25% TF loss over all time-points), and present in the structure, are identified by lilac beads. These sites were not selected, due to low TF loss. (**c**) Sites with two or more mutations among 385 CH505 Env sequences (at least 0.5% TF loss over all time-points), but less than 80% peak TF loss, are marked by lilac beads.

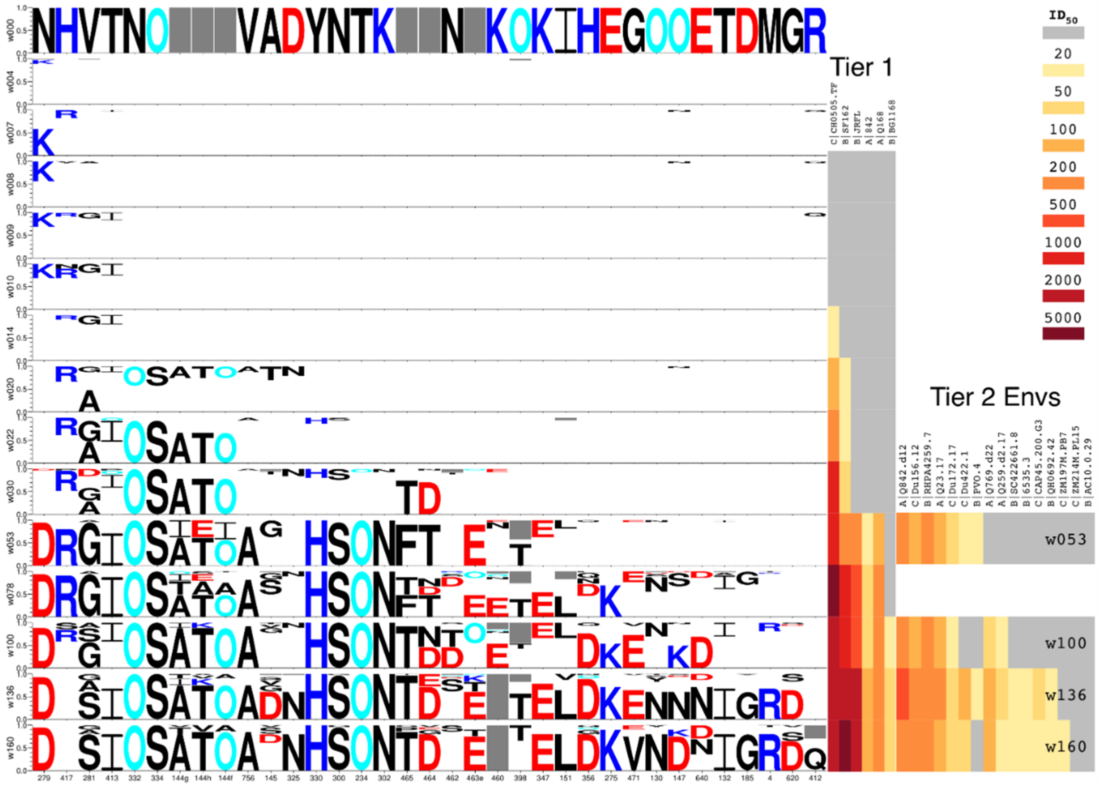

Figure S4. Comparative timing of TF loss and neutralization breadth. Sample times (weeks post-infection) increase from top to bottom, and align sequence logos (left) from **Figure 5b** with neutralization titers from contemporaneous plasmas tested against autologous virus (CH0505.TF), a panel of five Tier 1 envelope pseudotyped viruses (SF162 through BG1168), and sixteen Tier 2 viruses (Q842.d12 through AC10.0.29). ID_{50} titers below the sensitivity limit of 20 reciprocal dilutions are indicated by grey, and darker red colors indicate greater neutralization potency. The clade (A, B, or C) appears before each virus name.

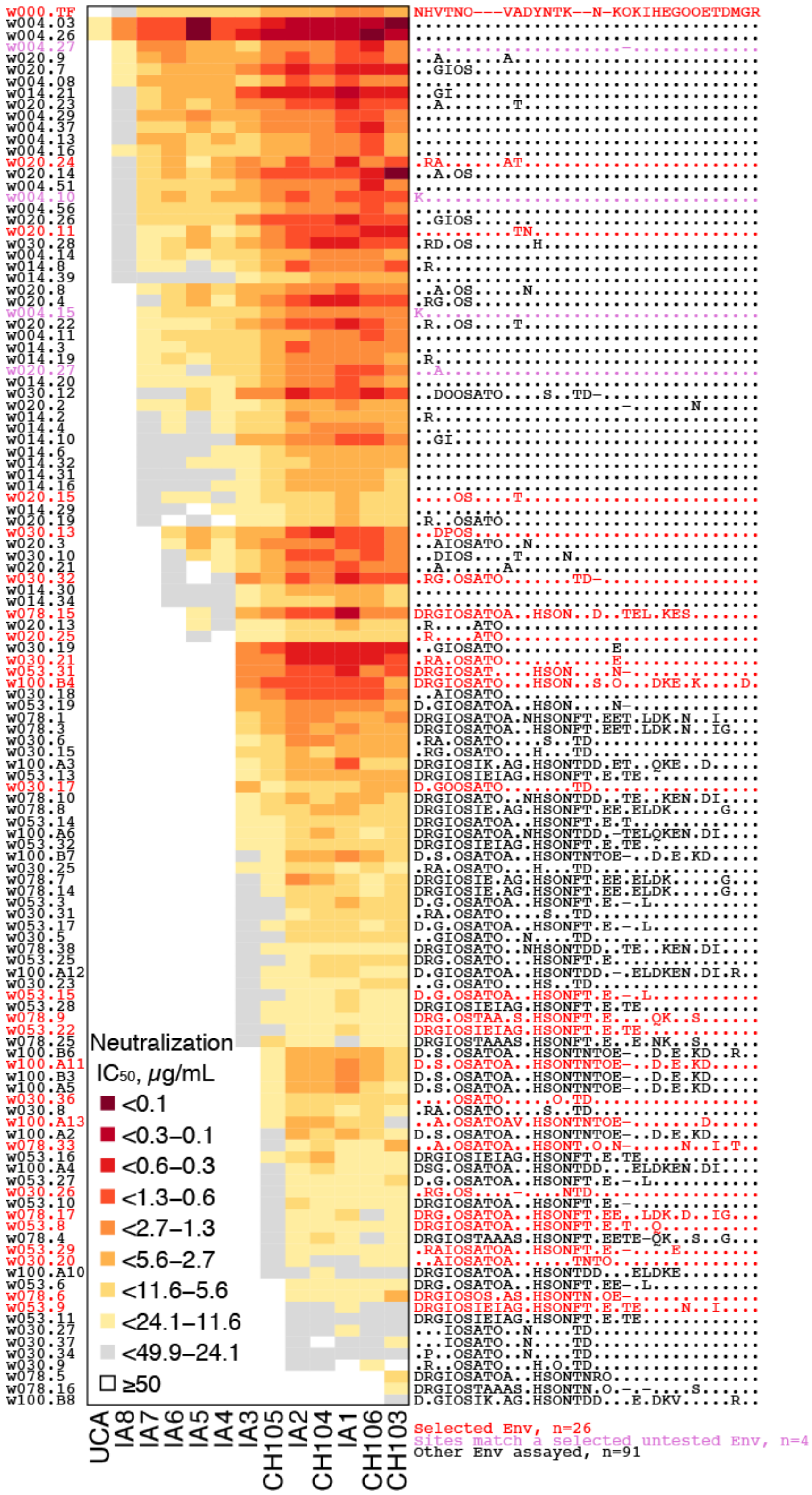

Figure S5. Selected Envs represent diverse neutralization phenotypes. Among the swarm of 54 Envs, 26 were cloned into pseudovirus backbones for TZM-bl neutralization assays (red text). Another four of the Env-pseudotyped virus constructs tested contained selected sites that matched with those in selected Envs (purple text). Neutralization IC_{50} titers are represented as colors to indicate sensitivity of each virus to neutralization by each mAb in the CH103 lineage. Selected Env sites correspond to concatamers listed in Table S3. Data values appear in Table S5.

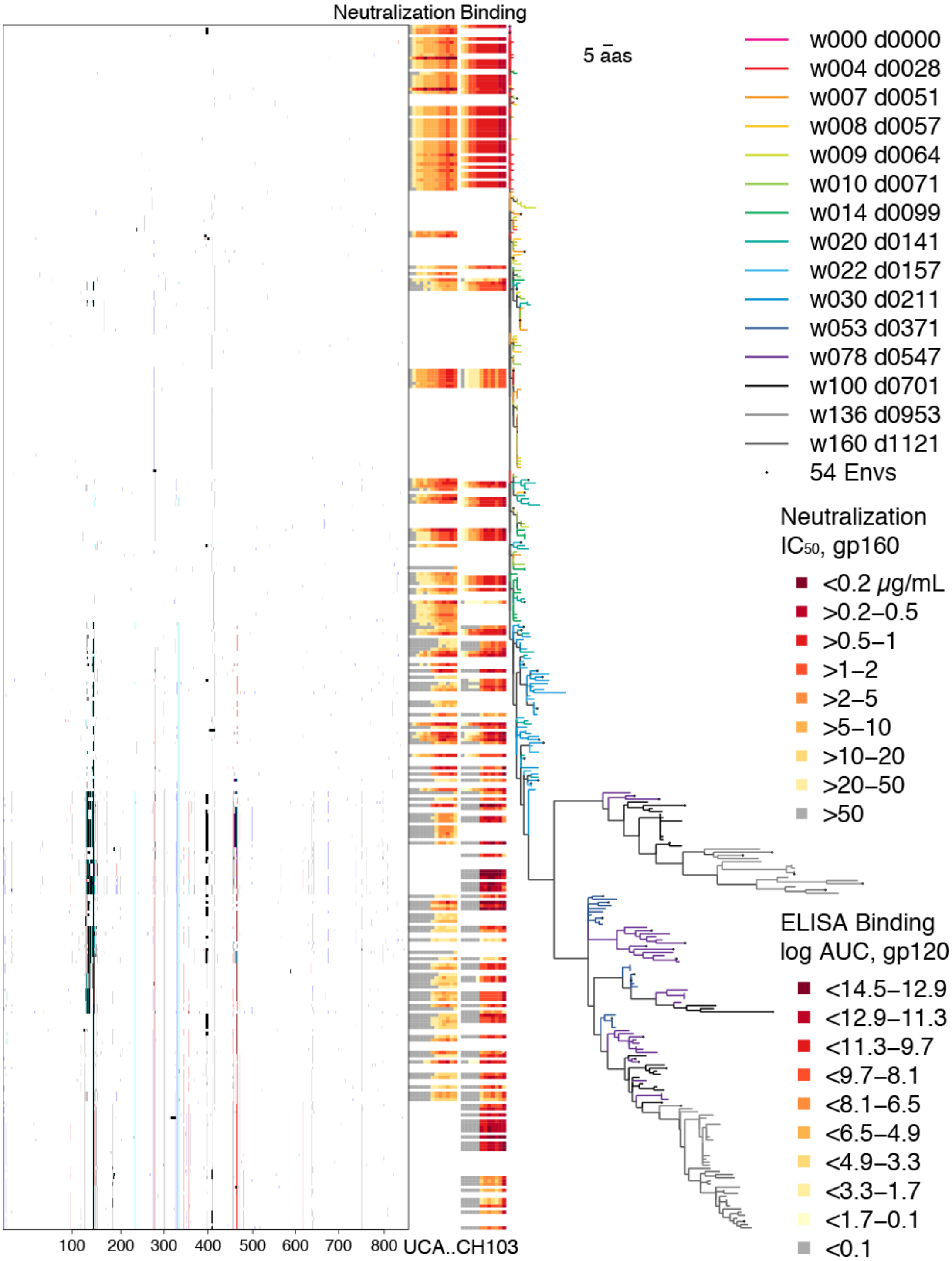

Figure S6. CH505 Env genotypes, phenotypes, and phylogeny. A pixel plot (left) is paired with heatmaps (center) of neutralization and binding assay results, and the phylogeny (right). Each row depicts one Env. Following Figure 7, this adds heatmaps of neutralization IC_{50} titers and ELISA log-AUC binding affinities of hand-picked envelopes from assays against the CH103 bnAb lineage. Data appear in Tables S4 and S5. Column order for bnAb titers follows that shown in Figures 8 and S5.

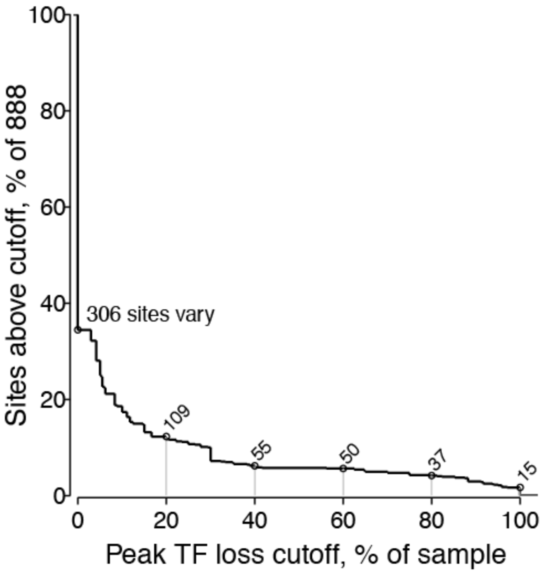

Figure S7. Number of sites varied with cutoff in chronically infected donor CH0457. Increasing the cutoff decreased number of sites.

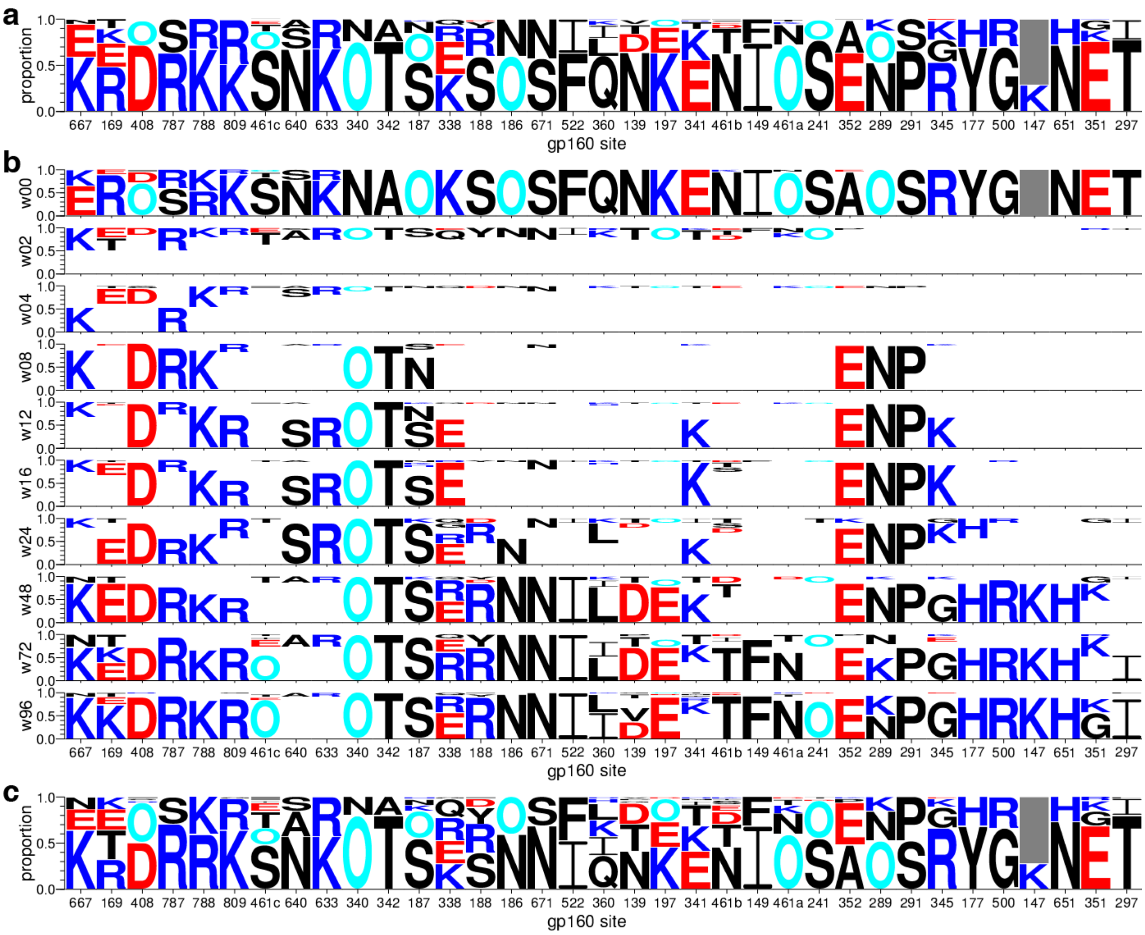

Figure S8. Variant frequencies among selected sites in chronic infection. Frequencies from CH0457, computed among (**a**) all sequences, pooled; (**b**) sequences stratified by time; and (**c**) 44 selected Envs. Colors indicate positive (blue) and negative (red) charges and "O" indicates a potentially gylcosylated asparagine (cyan). Indels appear as grey boxes.

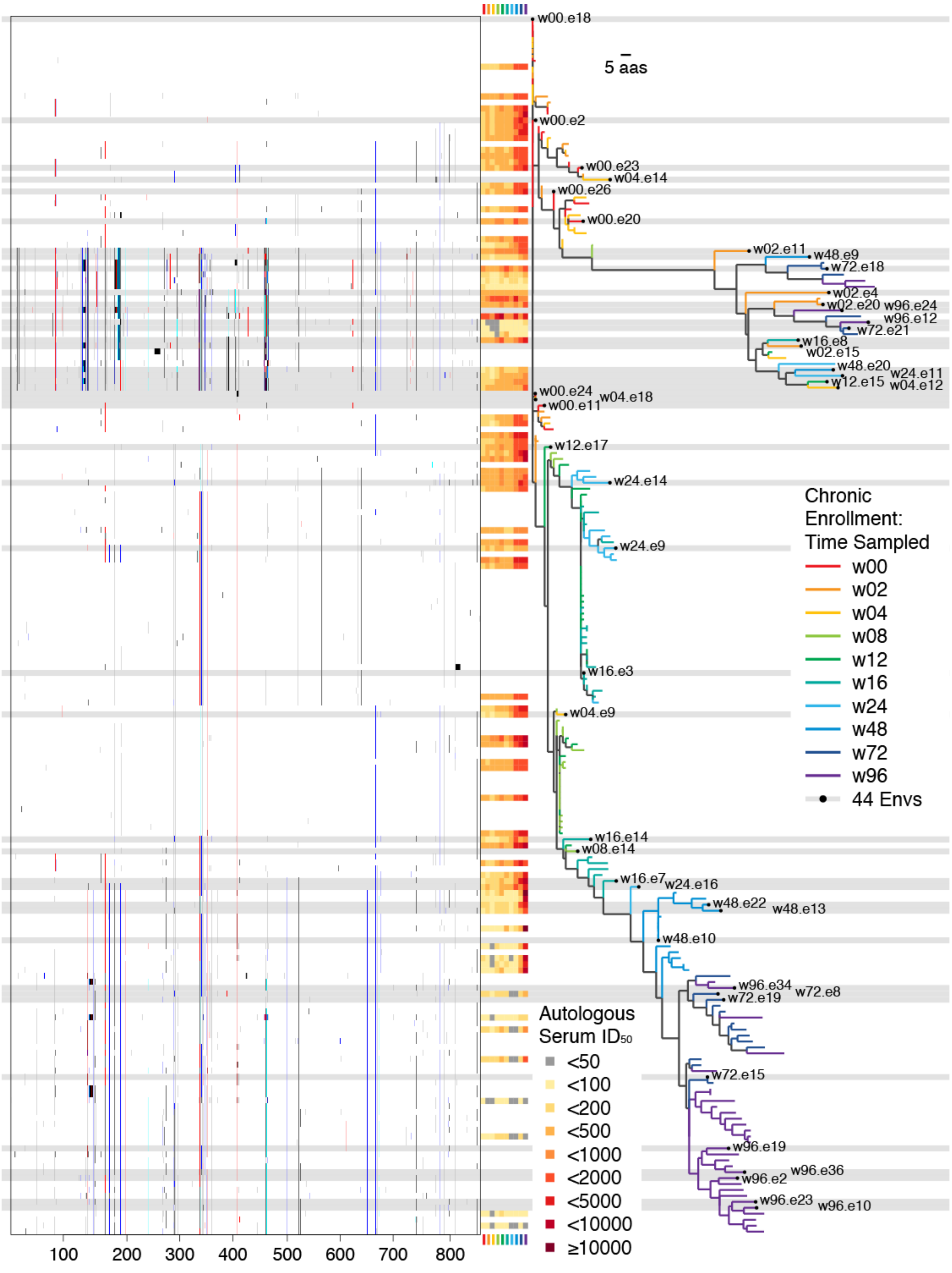

Figure S9. Diversity in chronic infection. CH0457 Env mutations (left), neutralization titers against autologous contemporaneous plasmas (center), and maximum-likelihood Env phylogeny (right). The 44 selected Envs are emphasized. The divergent clade appears above the "Time Sampled" legend. This representation follows Figure S6, and shows plasma neutralization as one column per time-point.

Table S1. V1 hypervariable region. This table shows variants in HXB2 positions 136 through 145, codons 199–215 in the CH505 alignment, a region not present in the transmitted-founder virus. This region accepted many distinct insertions, up to 17 amino acids long, often followed by deletions within the insertion, or subsequent mutations, throughout the period of the infection sampled. We found 29 distinct forms in this region. An example of each unique sequence selected from the first time-point in which each pattern was observed, with a representative sequence named, which includes the time of isolation, in weeks post-infection. The V1 loop noted in the Los Alamos database spans positions HXB2 132–153. In subject CH505 indels in this broader region are almost certainly also impacting evolutionary patterns. Dashes maintain alignment consistency.

Example Sequence	V1 Insertion	Number of Times Observed
w000.TF	-----------------	211
w020.19	AS------------NAT	19
w020.28	ASN----------TNAT	3
w022.10	--------------NAT	74
w022.17	-------------TNAT	12
w030.18	A-------------NAT	11
w030.33	TSNSS------------	1
w030.19	AR---------NCTNAT	1
w030.32	ASNSSI-----NCTNAT	1
w053.2	ASNATAS-------NAT	5
w053.22	A---TASNS----SIIE	11
w053.13	ASNATASNS----SIIE	3
w078.42	ASNATASNATAS---NS	2
w078.34	ASNATASNATAS-NTTA	1
w078.16	T-NATASNATAS-NATA	2
w078.36	ASNATASNATAS-NATA	7
w078.5	ATASNTNATAS-NINAT	12
w078.33	ATAS----------NAT	1
w100.A3	ATASNSSI-------IK	3
w100.T2	ATAS--------NINAT	3
w100.A13	ATASNANATAS-NTNAT	2
w136.B7	D----ANATASNTNATV	1
w136.B2	ATASNTNATVSNIKATV	1
w136.B3	ATASNANATASNTNATV	1
w136.B24	ATASNA--TASNTNATA	1
w136.B23	DTASNSSI-------IK	3
w160.C1	ANATASNI------NVT	1
w160.C12	ATASNANATVSNTNATV	3
w160.D6	--------------NTT	1

Table S2. V5 hypervariable region. This table summarizes variants in HXB2 positions 463 through 466, spanning codons 546–552 in the CH505 alignment, a region that was not present in the transmitted-founder virus. This region accepted many distinct insertions, up to 7 amino acids long, often followed by deletions within the insertion, or subsequent mutations, throughout the period of the infection sampled. We found 21 distinct forms of indels in this region. We selected an example of each unique sequence from the time-point in which each pattern was first observed, and a representative sequence named, which includes the week of isolation. The V5 loop region noted in the Los Alamos database spans HXB2 positions 460–467. In subject CH505, indels in this broader region are almost certainly also impacting the evolutionary patterns. Dashes maintain the alignment.

Example Sequence	V5 Insertion	Number of Times Observed
w000.TF	-------	241
w030.11	-----DT	40
w030.20	DGGKNNT	1
w053.15	----ETF	40
w078.42	-GGKNNT	5
w078.16	--GKNNT	1
w078.29	-----NT	1
w078.33	------T	1
w078.5	-RGKNNT	1
w100.B7	DGGNNNT	11
w136.B1	----EDT	38
w136.B23	----TET	5
w136.B5	----KET	4
w136.B26	----NDT	1
w136.B28	---DTDT	1
w136.B2	-----DI	1
w136.B7	-----DM	1
w160.C3	-----DP	2
w160.C10	ETSETVS	1
w160.C1	----TGT	1
w160.C6	----EGT	1

Table S3. Selected Envs. Concatamers (35 sites with at least 80% TF loss) in antigenic swarm of 54 Envs, selected to represent polymorphisms among 385 Env gp160s from CH505. Dots match TF state and dashes are place-holders for deletions.

Name	Accession	Concatamer
w000.TF	KC247556	NHVTNO---VADYNTK--N-KOKIHEGOOETDMGR
w004.31	KC247583	.....................-.............
w004.54	KC247604	K..................................
w007.8	KM284749	KR.................................
w007.21	KM284732	..................................Q
w007.25	KM284734	............................N......
w007.34	KM284744	...I...............................
w008.20	KM284762	..A................................
w009.19	KM284781	..G................................
w010.7	KM284714	.N.................................
w020.15	KC247489	....OS....T........................
w020.11	KC47485	..........TN.......................
w020.24	KC247495	.RA......AT........................
w020.25	KC247496	.R....ATO..........................
w022.6	KC247523	..AIOSATO...H......................
w022.5	KC247522	.RA.OSATO....S.....................
w022.9	KC247525	..GIOS.................-...........
w022.22	KM284717	...O...............................
w030.20	KC247541	..AIOSATOA......TNTO...............
w030.17	KC247532	D.GOOSATO.......TD.................
w030.21	KC247535	.RA.OSATO...........E..............
w030.36	KC247549	....OSATO.....O.TD.................
w030.26	KC247539	.RG.OS....-....NTD.................
w030.13	KC247529	..DPOS.............................
w030.32	KC247546	.RG.OSATO.......TD-................
w053.15	KC247614	D.G.OSATOA..HSONFT.E.-.L...........
w053.29	KC247625	.RAIOSATOA..HSONFT.E.-....E........
w053.22	KC247620	DRGIOSIEIAG.HSONFT.E.TE............
w053.8	KC247632	DRGIOSATOA..HSONFT.E.T..Q..........
w053.31	KC247628	DRGIOSAT....HSON....N-.............
w053.9	KC247633	DRGIOSIEIAG.HSONFT.E.TE....N..I....
w078.6	KC247664	DRGIOSOS.AS.HSONTN.OE-.............
w078.36	KC247655	DRGIOSTAAAS.HSON..S.O-.-....SD.....
w078.9	KC247667	DRG.OSTAA.S.HSONFT.E....QK..S......
w078.26	KC247645	DRGIOSTAAAS.HSON..S.O.E.NK..S......
w078.29	KC247647	DRGIOSTAAAS.HSONTN..-..-L..NS.A....
w078.30	KC247649	..A.OSATOA..HSON....N-......D......
w078.33	KC247652	..A.OSATOA..HSONT.O.N-.....N..I.T..
w078.17	KC247639	DRG.OSATOA..HSONFT.EE..LDK.D..IG...
w078.15	KC247637	DRGIOSATOA..HSON..D..TEL.KES.......
w078.27	KC247646	DRGIOSATOA..HSONTDD..TEL.KES....R..
w100.T3	KC247401	DRGIOSATO..NHSONTDD.ETEL.KEN..I.RS.
w100.B10	KC247386	DSG.OSATOA..HSONTDD....LDKEN..I....
w100.B2	KC247387	.RAIOSIK.AG.HSON....N...D.V........
w100.B4	KC247389	DRGIOSATO...HSON..S.O...DKE.K....D.
w100.A11	KC247376	D.S.OSATOA..HSONTNTOE-..D.E.KD.....
w100.A13	KC247378	..A.OSATOAV.HSONTNTOE-.......D.....

Table S3. *Cont.*

Name	Accession	Concatamer
w136.B10	KC247404	D.GIOSATOADNHSONTD.E-TELDKES.DIY.S.
w136.B5	KC247429	..A.OSATOAV.HSONTESK-.E.O..Y.DI....
w136.B2	KC247411	D.G.OSTVAA-.HGONIDOT--E.O.......RD.
w136.B23	KC247414	D.A.OSIK..G.HSONTEST-..VD....N...D.
w160.C1	KC247465	..A.OSVTOAV.HSONTGST-...D..Y.D..TV.
w160.T3	KC247482	D.SIOSATOA.NHSONTD.E-TELDKVND.IGRD-
w160.T4	KC247483	D.A.OSTVA.S.HSONPD..-...G...DN.....

Table S4. ELISA binding assay log-AUC of CH103 lineage Abs against autologous Env gp120s.

gp120	UCA	IA8	IA7	IA6	IA4	IA3	CH105	IA2	CH104	IA1	CH106	CH103
**w000.TF**	3.5	5.5	9.2	9.1	10.1	11	11.2	10.8	10.4	10.4	11.3	12.6
**w004.26**	3.2	5.5	9.1	9	9.5	10.4	10.4	10.6	11.1	10.3	11	12.4
**w004.54**	<0.1	0.5	2.3	2.9	2.8	5.1	8.3	6.8	8.1	6.2	8.1	9.2
**w014.10**	0.3	2.2	5.1	6.3	7.1	9	10.8	9.9	10.7	9.4	10.4	11.9
**w014.2**	0.8	3.2	7.2	6.9	8.5	9.7	9.6	9.7	9.5	9.1	9.6	10.9
**w014.21**	0.4	2.8	6.2	7.8	8	9.4	11.4	10.2	11.4	9.7	11.1	13.1
**w014.3**	1	4.4	7.7	7.6	9.2	10.3	10.8	10.6	10.6	9.6	10.6	11.9
**w014.32**	1.6	4.2	7.9	7.8	8.5	9.9	10.7	10.2	10.9	9.5	10.5	11.9
**w014.8**	<0.1	0.9	0.6	2.4	2.6	1.8	6	3.6	3.9	3.4	7.9	11.2
**w020.11**	<0.1	0.9	0.1	0.8	0.8	0.8	3.6	2.6	2.2	1.8	5.4	9.6
**w020.14**	0.3	3.4	7.2	7.9	8.6	9.5	10.4	11	10.4	10.3	11.2	12.6
**w020.15**	1.6	4.2	8.2	7.8	9.1	10.2	10.8	10.5	10.5	9.9	10.5	11.8
**w020.19**	0.8	3.5	7.2	6.5	8.1	9.4	9.5	9.3	9.7	9	9.7	11.3
**w020.22**	1.2	4	8.3	8.4	8.8	10.2	10.5	10.6	10.7	10.1	10.7	11.5
**w020.23**	0.9	3.6	7.2	7.9	8.6	9.7	11.2	10.7	11.6	9.8	11.3	12.8
**w020.25**	0.8	2.4	6.4	6	7.3	8.6	8.2	9	8.3	8.6	9.4	10.3
**w020.26**	<0.1	0.7	2.7	4	5.1	7.1	9	9	8.7	8.9	9.6	11.3
**w020.3**	0.8	2.6	5.9	6	7	7.7	10.1	9.3	9.8	8.9	10.3	12.4
**w020.30**	0.8	4.2	7.3	7.9	8.5	9	10.3	10.5	10.1	9.8	10.8	11.7
**w020.4**	0.9	3.6	8.2	8.7	9.4	10.6	11.1	11.6	11.6	11.3	11.5	12.5
**w020.7**	0.6	2.7	7.6	8.1	8.7	10.4	10.6	11.3	11.1	10.8	11.3	12.5
**w020.8**	0.4	2.6	5.9	6.7	7.4	8.8	10.6	10.5	10.5	9.9	11	12.4
**w020.9**	0.4	2.8	4.9	6.8	7.4	8.1	9.3	9.7	8.9	9	10	11.9
**w030.10**	0.2	1.3	3.2	5.1	5.8	7.3	9.3	11	10.3	10.1	11.1	12.5
**w030.11**	<0.1	<0.1	0.7	0.5	0.5	2.4	9	9.1	9.5	8.4	9.4	10.9
**w030.12**	<0.1	0.4	1	1.2	1.9	4.1	10.7	11.6	11.6	10.6	11.8	13.3
**w030.13**	0.3	2	4.7	6.5	7.4	9	10.5	11.4	11.3	10.5	11.9	12.9
**w030.15**	<0.1	<0.1	<0.1	<0.1	<0.1	<0.1	9.3	8.4	9.7	8	9.3	10.1

Table S4. *Cont.*

gp120	UCA	IA8	IA7	IA6	IA4	IA3	CH105	IA2	CH104	IA1	CH106	CH103
**w030.17**	<0.1	<0.1	<0.1	<0.1	<0.1	<0.1	6.7	7	7	5.8	8.2	9.9
**w030.18**	<0.1	<0.1	<0.1	<0.1	<0.1	<0.1	11.1	9.4	10.9	9.9	10.5	13
**w030.19**	<0.1	<0.1	<0.1	<0.1	<0.1	<0.1	9.5	8.2	8.9	8.2	7.4	12.9
**w030.20**	<0.1	<0.1	<0.1	<0.1	<0.1	<0.1	7.1	6.3	7.5	5.7	7.3	9.5
**w030.21**	<0.1	<0.1	<0.1	<0.1	<0.1	<0.1	10.6	11.5	11.3	11.8	10.9	12.2
**w030.23**	<0.1	<0.1	<0.1	<0.1	<0.1	0.7	9.2	8.9	9.6	8.4	10	11.1
**w030.25**	<0.1	<0.1	0.4	0.5	0.4	1.4	10	9.9	10.3	9.1	10.2	12.5
**w030.27**	<0.1	<0.1	<0.1	<0.1	<0.1	0.4	7.9	7.9	8.2	7.3	8.2	9.1
**w030.28**	<0.1	1.6	3.5	6.3	6.5	7.7	9.1	11.1	10.7	10.1	11.7	12.8
**w030.36**	<0.1	<0.1	<0.1	<0.1	<0.1	<0.1	7.5	7.3	7.8	6.7	8.5	9.1
**w030.5**	<0.1	<0.1	<0.1	<0.1	<0.1	0.3	9	8.1	9.4	7.8	9.4	11.4
**w030.6**	<0.1	<0.1	<0.1	<0.1	<0.1	<0.1	6.5	5.4	6.1	4.5	6.6	8
**w030.9**	<0.1	<0.1	<0.1	<0.1	<0.1	0.2	8.7	8.6	8.9	7.8	9.6	10.2
**w053.13**	<0.1	<0.1	<0.1	<0.1	<0.1	<0.1	9.4	10.5	10	9.9	10.2	11.2
**w053.15**	<0.1	<0.1	<0.1	<0.1	<0.1	<0.1	9.4	9.5	10.4	9.2	10.1	11.2
**w053.19**	<0.1	<0.1	<0.1	<0.1	<0.1	<0.1	13.8	12.6	13.8	13.6	13.2	13.5
**w053.22**	<0.1	<0.1	<0.1	<0.1	0.2	1.1	9	9.3	9.9	8.8	9.8	11.6
**w053.25**	<0.1	<0.1	<0.1	<0.1	<0.1	<0.1	10	10.2	10.6	10	10.9	11.6
**w053.29**	<0.1	<0.1	<0.1	<0.1	<0.1	<0.1	8.5	9.2	9.8	8.8	9.7	10.2
**w053.31**	<0.1	<0.1	<0.1	<0.1	<0.1	<0.1	13.5	13.3	13.7	13.4	13.4	13.6
**w053.6**	<0.1	<0.1	<0.1	<0.1	<0.1	<0.1	11.7	11.9	12	11.4	10.9	12.7
**w078.1**	<0.1	<0.1	<0.1	<0.1	<0.1	<0.1	7.5	7.7	8.3	7.5	7.3	11
**w078.10**	<0.1	<0.1	<0.1	<0.1	<0.1	<0.1	7.6	6.6	8.1	6.6	7.8	11.1
**w078.15**	<0.1	<0.1	0.7	1	1.3	3	10.1	11.5	10.8	10.9	11	10.7
**w078.17**	<0.1	<0.1	<0.1	<0.1	<0.1	<0.1	9.5	9.9	10	9	8.7	11.3
**w078.25**	<0.1	<0.1	<0.1	<0.1	<0.1	<0.1	3.3	3.2	3.5	2.4	4.6	8.7
**w078.33**	<0.1	<0.1	<0.1	<0.1	<0.1	<0.1	8.9	9	9	8.2	9.5	11.1
**w078.38**	<0.1	<0.1	<0.1	<0.1	<0.1	<0.1	4	4.7	4.2	4.4	5.3	7.7

Table S4. *Cont.*

gp120	UCA	IA8	IA7	IA6	IA4	IA3	CH105	IA2	CH104	IA1	CH106	CH103
**w078.4**	<0.1	<0.1	<0.1	<0.1	<0.1	<0.1	9.5	10.2	10.1	9.8	9.2	11.7
**w078.5**	<0.1	<0.1	<0.1	<0.1	<0.1	<0.1	6.6	6.6	7.7	5.6	7.9	10.2
**w078.6**	<0.1	<0.1	<0.1	<0.1	<0.1	<0.1	3.8	4.6	4.5	3	6.7	9.9
**w078.7**	<0.1	<0.1	<0.1	<0.1	<0.1	<0.1	9.4	9.2	9.5	8.9	8.7	11.4
**w078.9**	<0.1	<0.1	<0.1	<0.1	<0.1	<0.1	6.6	7.2	6.9	6.3	8.3	8.9
**w100.A10**	<0.1	<0.1	<0.1	<0.1	<0.1	0.1	9.1	10.5	10.1	9.9	10.4	11.7
**w100.A12**	<0.1	<0.1	<0.1	<0.1	<0.1	<0.1	9.6	9.4	10.4	8.6	9.7	4.7
**w100.A13**	<0.1	<0.1	<0.1	<0.1	<0.1	<0.1	11.4	12.5	12.9	12.6	12	12.9
**w100.A3**	<0.1	<0.1	<0.1	<0.1	<0.1	<0.1	7.2	9.3	10	8.5	8.6	11.4
**w100.A4**	<0.1	<0.1	<0.1	<0.1	<0.1	<0.1	8.1	9	9.1	8.6	9	10.7
**w100.A6**	<0.1	<0.1	<0.1	<0.1	<0.1	<0.1	10.1	11.4	11.6	11	11.1	12.1
**w100.B2**	<0.1	<0.1	<0.1	<0.1	<0.1	<0.1	7.5	7.5	6.1	7.3	7.8	3.2
**w100.B4**	<0.1	<0.1	<0.1	<0.1	<0.1	<0.1	11.9	13.4	13.1	13.7	12.6	9.7
**w100.B6**	<0.1	<0.1	<0.1	<0.1	<0.1	<0.1	11	12.1	11	12.2	11.8	7.1
**w100.B7**	<0.1	<0.1	<0.1	<0.1	<0.1	<0.1	11.6	12.4	11.6	11.9	11.5	7.8
**w100.C7**	<0.1	<0.1	<0.1	<0.1	<0.1	<0.1	9	9.1	9.2	8.9	9.3	4.8
**w136.B10**	<0.1	<0.1	<0.1	<0.1	<0.1	<0.1	8.5	9.7	8.9	9.2	9	11.5
**w136.B12**	<0.1	<0.1	<0.1	<0.1	<0.1	<0.1	9.7	12.4	10.7	12.2	11.9	12.7
**w136.B2**	<0.1	<0.1	<0.1	<0.1	<0.1	<0.1	12.4	13.1	13.2	13.2	12.7	10.8
**w136.B20**	<0.1	<0.1	<0.1	<0.1	<0.1	<0.1	10.7	12.7	11.7	12.4	12.1	12.4
**w136.B23**	<0.1	<0.1	<0.1	<0.1	<0.1	<0.1	13.7	14.3	14.2	14.4	13.3	11.8
**w136.B27**	<0.1	<0.1	<0.1	<0.1	<0.1	<0.1	10.4	11.9	11.5	11.2	11.2	12.6
**w136.B29**	<0.1	<0.1	<0.1	<0.1	<0.1	<0.1	10.2	12.5	11.4	12.2	12	12.7
**w136.B3**	<0.1	<0.1	<0.1	<0.1	<0.1	0.1	12	13.1	13.1	13.1	12.6	9.9
**w136.B36**	<0.1	<0.1	<0.1	<0.1	<0.1	<0.1	12.8	13.6	13.8	13.8	13.1	13.7
**w136.B4**	<0.1	<0.1	<0.1	<0.1	<0.1	<0.1	10.7	12.2	12.2	11.8	11.8	13.4
**w136.B5**	<0.1	<0.1	<0.1	<0.1	<0.1	<0.1	10.5	9.9	10.6	9.5	10.9	4.3
**w136.B8**	<0.1	<0.1	<0.1	<0.1	<0.1	<0.1	9.9	13.2	11.7	12.9	11.7	11.9

Table S4. *Cont.*

gp120	UCA	IA8	IA7	IA6	IA4	IA3	CH105	IA2	CH104	IA1	CH106	CH103
**w160.A1**	<0.1	<0.1	<0.1	<0.1	<0.1	<0.1	6.7	7.6	7.3	6.4	7.6	10.7
**w160.C11**	<0.1	<0.1	<0.1	<0.1	<0.1	<0.1	7.7	8.5	7.4	7.7	8.2	11.9
**w160.C12**	<0.1	<0.1	<0.1	<0.1	<0.1	<0.1	11.1	12.9	11.3	12.9	12.2	7.8
**w160.C14**	<0.1	<0.1	<0.1	<0.1	<0.1	<0.1	6.6	7	6.8	5.8	6.9	12.1
**w160.C2**	<0.1	<0.1	<0.1	<0.1	<0.1	1.3	8.6	10.1	9.2	9.6	9.3	12.2
**w160.C4**	<0.1	<0.1	<0.1	<0.1	<0.1	<0.1	6.6	6.2	7.2	5.3	6.8	12.2
**w160.D1**	<0.1	<0.1	<0.1	<0.1	<0.1	<0.1	6	7.3	5.7	6.4	7.1	11.5
**w160.D5**	<0.1	<0.1	<0.1	<0.1	<0.1	<0.1	4.6	5	4.5	3.8	5.6	10.9
**w160.T2**	<0.1	<0.1	<0.1	<0.1	<0.1	<0.1	4.9	5.6	5.4	4.7	6.2	11.2
**w160.T4**	<0.1	<0.1	<0.1	<0.1	<0.1	<0.1	12.2	13.4	12.8	13.6	12.3	9

Table S5. TZM-bl neutralization IC_{50} titers of CH103 lineage Abs with autologous *env* gp160s.

gp160	UCA	IA8	IA7	IA6	IA5	IA4	IA3	CH105	IA2	CH104	IA1	CH106	CH103
**w000.TF**	>50	44.67	10.17	10.17	6.82	9.72	6.62	5.1	2.73	3.08	1.42	2.29	4.14
**w004.03**	15.12	2.05	0.71	0.99	0.13	0.67	0.85	0.38	0.29	0.29	0.16	0.16	0.13
**w004.08**	>50	16.75	4.2	4.11	3.38	3.24	6.09	2.92	1.29	1.62	0.95	1.39	1.89
**w004.10**	>50	41.36	8.66	5.32	5.66	3.8	3.68	2.44	1.17	1.31	0.74	0.71	1.1
**w004.11**	>50	>50	14.35	10.32	8.17	8.76	7.71	3.62	2.79	2.32	1.71	1.2	2.37
**w004.13**	>50	25.24	3.08	3.5	9.78	4.08	7.88	3.4	3.46	2.09	1.45	1.08	3.15
**w004.14**	>50	47.61	11.92	9.71	13.78	11.02	10.62	5.22	3.53	2.34	2.14	1.32	4
**w004.15**	>50	>50	16.45	9.82	6.43	8.2	6.23	4.03	1.92	1.49	1.34	1.41	1.37
**w004.16**	>50	21.26	8.43	4.33	7.32	6.95	6.4	4.33	2.78	2.66	1.39	1.23	2.83
**w004.26**	19.5	1.5	0.77	0.88	0.07	0.67	0.41	0.24	0.15	0.27	0.25	0.09	0.24
**w004.27**	>50	12.39	2.32	2.57	4.69	2.7	5.7	2.4	1.89	1.44	1.17	0.53	1.94
**w004.29**	>50	31.68	5.57	4.72	2.48	4.7	3.4	2.24	1.3	1.36	1.1	0.79	1.9
**w004.37**	>50	25.63	8.66	7.97	4.76	5.61	4.15	2.19	1.68	1.65	1	0.43	1.87
**w004.51**	>50	34.15	6.83	5.77	8.62	6.54	5.45	2.11	1.54	1.59	1.36	0.56	2.04
**w004.56**	>50	34.78	8.46	8.51	6.42	6.35	5.83	3.36	2	1.59	1.39	0.8	2.4
**w014.10**	>50	>50	28.28	29.42	25.98	28.93	4.98	2.82	1.72	1.9	1.27	0.98	1.6
**w014.16**	>50	>50	42.21	27.23	32.29	18.85	11.75	8.42	4.3	4.48	4.08	4.45	6.27
**w014.19**	>50	>50	15.49	10.14	20.09	9.2	6.06	4.39	1.82	2.47	1.36	2.47	4.66
**w014.2**	>50	>50	26.66	14.92	25.33	13.63	10.3	6.84	2.28	3.24	2.87	2.86	4.01
**w014.20**	>50	>50	23.56	12.68	20.3	15.94	6.27	3.02	1.96	1.88	1.07	1.43	2.92
**w014.21**	>50	24.97	11.01	5.22	4.07	3.36	0.94	0.6	0.39	0.37	0.24	0.41	0.53
**w014.29**	>50	>50	37.65	39.45	>50	22.36	14.88	12.54	5.66	6.14	4.02	6.33	6.91
**w014.3**	>50	>50	23.5	13.51	8.13	10.07	5.83	3.64	1.18	1.69	1.84	1.45	2.57
**w014.30**	>50	>50	>50	46.59	39.45	34.46	15.52	8.11	4.94	4.2	3.01	3.41	6.17
**w014.31**	>50	>50	36.38	25.99	31.67	28.03	13.04	7.22	4.05	3.69	4.28	3.19	5.84
**w014.32**	>50	>50	41.85	25.02	21.56	22.67	16	8.26	5.66	3.96	3.66	4.41	5.51
**w014.34**	>50	>50	>50	43.94	48.04	49.79	22.64	11.24	7.05	5.72	5.09	4.66	7.32
**w014.39**	>50	43.27	40.99	24.91	34.07	32.87	15.15	8.48	5.77	4.73	3.09	4.52	5.44
**w014.4**	>50	>50	24.72	20.23	28.95	15.59	10.41	6.04	2.77	2.35	2.25	2.55	3.37

Table S5. *Cont.*

gp160	UCA	IA8	IA7	IA6	IA5	IA4	IA3	CH105	IA2	CH104	IA1	CH106	CH103
**w014.6**	>50	>50	26.84	25.13	32.85	20.88	12.23	6.99	3.37	3.14	3.07	3.02	4.14
**w014.8**	>50	28.47	23.12	17.3	34.15	12.93	8.81	4.59	1.06	1.41	1.54	1.6	1.06
**w020.11**	>50	32.68	21.45	13.04	4.41	12.53	5.97	1.82	1.14	0.85	1.17	0.61	0.43
**w020.13**	>50	>50	>50	>50	19.84	31.77	20.17	14.16	6.7	5.6	3.62	6.15	5.82
**w020.14**	>50	42.47	10.13	5.45	6.33	4.88	1.99	1.04	1.22	0.67	0.72	0.46	0.08
**w020.15**	>50	>50	42.09	21	19.46	29.54	16.25	11.42	6.41	5.9	3.12	6.16	6.91
**w020.19**	>50	>50	43.58	>50	43.43	>50	21.65	13.42	7.58	6.12	5.05	7.69	8.34
**w020.2**	>50	>50	20.17	18.4	7.09	19.49	11.8	6.2	4.08	3.29	2.12	2.78	3.5
**w020.21**	>50	>50	>50	29.58	>50	21.02	6.36	3.64	1.88	1.81	1.23	1.56	2.54
**w020.22**	>50	>50	20.77	12.38	14.1	8.22	3.48	2.21	1.25	0.88	0.53	1.01	1.45
**w020.23**	>50	26.79	8.07	4.74	10.6	3.45	2.65	1.48	1.12	0.87	0.54	0.68	0.87
**w020.24**	>50	28.92	9.44	5.57	12.97	4.73	2.51	3.15	1.12	1.52	0.57	1.54	1.11
**w020.25**	>50	>50	>50	>50	37.72	>50	23.37	14	11.15	9.01	6.41	7.96	7.95
**w020.26**	>50	48.25	10.45	7.31	8.65	6.79	2.06	1.23	0.9	1.29	0.51	0.77	0.88
**w020.27**	>50	>50	20.82	14.21	25.37	9.51	5.7	3.89	2.24	1.85	1.2	1.22	1.81
**w020.3**	>50	>50	>50	24.06	4.17	22.41	4.93	2.35	1.63	1.46	0.77	1.04	1.41
**w020.4**	>50	>50	25.99	9.46	3.98	11.83	2.77	1.36	0.88	0.6	0.55	0.67	0.67
**w020.7**	>50	23.68	6.85	2.85	2.92	3.83	1.39	0.86	0.62	0.95	0.41	0.6	0.52
**w020.8**	>50	>50	14.67	8.41	3.78	7.53	3.34	3.54	1.37	1.63	1.14	1.7	2.02
**w020.9**	>50	14.87	5.46	2.07	4.3	3.06	3.62	1.92	1.16	1.33	1.06	1.13	1.37
**w030.10**	>50	>50	>50	35	6.93	23.97	8.07	3.88	1.11	1.08	1.61	1.14	1.56
**w030.12**	>50	>50	36.65	25.87	6.44	18.39	1.92	1.88	0.52	0.95	0.65	0.62	1.11
**w030.13**	>50	>50	>50	8.43	2.77	9.64	5.19	3.86	1	0.43	1.06	1.08	1.43
**w030.15**	>50	>50	>50	>50	>50	>50	11.44	7.43	3.5	5.79	4.6	2.8	3.98
**w030.17**	>50	>50	>50	>50	>50	>50	3.86	14.24	7.28	6.78	6.86	3.9	4.28
**w030.18**	>50	>50	>50	>50	>50	>50	2.92	2.1	1.12	1.16	1.08	0.88	1.36
**w030.19**	>50	>50	>50	>50	>50	>50	1.45	0.81	0.47	0.51	0.39	0.54	0.58
**w030.20**	>50	>50	>50	>50	>50	>50	>50	44.19	20.42	28.33	31.48	17.56	13.2

Table S5. *Cont.*

gp160	UCA	IA8	IA7	IA6	IA5	IA4	IA3	CH105	IA2	CH104	IA1	CH106	CH103
**w030.21**	>50	>50	>50	>50	>50	>50	2.35	1.49	0.43	0.53	0.62	0.56	1.05
**w030.23**	>50	>50	>50	>50	>50	>50	33.12	23.24	18.56	12.81	17.21	9.39	9.51
**w030.25**	>50	>50	>50	>50	>50	>50	20.19	12.84	11.02	8.69	12.9	7.7	11.99
**w030.26**	>50	>50	>50	>50	>50	>50	>50	32.38	17.23	18.9	12.5	12.48	12.11
**w030.27**	>50	>50	>50	>50	>50	>50	>50	>50	43.69	38.02	18.28	31.87	30.02
**w030.28**	>50	44	23.07	7.18	3.78	6.92	4.35	2.13	0.77	0.59	0.49	0.77	0.77
**w030.31**	>50	>50	>50	>50	>50	>50	43.08	27.73	11.73	9.79	6.71	10	8.79
**w030.32**	>50	>50	>50	33.9	>50	40.93	2.56	2.89	1.09	1.52	0.54	1.25	1.03
**w030.34**	>50	>50	>50	>50	>50	>50	>50	>50	44.85	47.07	49.72	39.83	26.37
**w030.36**	>50	>50	>50	>50	>50	>50	>50	21.15	10.96	11.07	8.12	8.13	9.24
**w030.37**	>50	>50	>50	>50	>50	>50	>50	>50	29.75	>50	25.19	40.42	22.3
**w030.5**	>50	>50	>50	>50	>50	>50	45.16	34.12	6.76	7.45	9.67	10.04	8.05
**w030.6**	>50	>50	>50	>50	>50	>50	15.27	9.23	1.3	1.3	4.99	4.51	4.15
**w030.8**	>50	>50	>50	>50	>50	>50	>50	14.11	10.35	7.07	12.83	9.91	12.56
**w030.9**	>50	>50	>50	>50	>50	>50	>50	>50	44.49	42.98	>50	19.82	49.94
**w053.10**	>50	>50	>50	>50	>50	>50	>50	31.68	10.59	20.07	13.21	12.73	18.87
**w053.11**	>50	>50	>50	>50	>50	>50	>50	>50	26.22	32.63	32.31	37.41	30.37
**w053.13**	>50	>50	>50	>50	>50	>50	12.38	6.64	4.78	4.47	3.6	4.66	5.3
**w053.14**	>50	>50	>50	>50	>50	>50	20.93	15.52	6	6.46	5.76	8.28	8.01
**w053.15**	>50	>50	>50	>50	>50	>50	31.54	29.48	12.11	12.65	8.19	17.72	13.82
**w053.16**	>50	>50	>50	>50	>50	>50	>50	21.73	10.68	3.44	17.37	18.39	12.31
**w053.17**	>50	>50	>50	>50	>50	>50	43.24	20.89	10.62	7.25	10.32	13.8	10.61
**w053.19**	>50	>50	>50	>50	>50	>50	4.58	2.84	2.59	2.66	1.91	2.55	3.16
**w053.22**	>50	>50	>50	>50	>50	>50	41.2	29.51	15.81	12.31	11.58	19.36	12.19
**w053.25**	>50	>50	>50	>50	>50	>50	43.14	28.05	13.07	10.1	7.56	10.47	9.26
**w053.27**	>50	>50	>50	>50	>50	>50	>50	27.02	17.47	13.61	14.56	14.99	10.47
**w053.28**	>50	>50	>50	>50	>50	>50	46.24	21.84	14.19	14.45	7.67	15.86	12.65
**w053.29**	>50	>50	>50	>50	>50	>50	>50	42.24	21.79	17.38	14.58	22.52	21.95
**w053.3**	>50	>50	>50	>50	>50	>50	49.31	27.54	10.51	8.82	4.42	9.46	8.37

Table S5. *Cont.*

gp160	UCA	IA8	IA7	IA6	IA5	IA4	IA3	CH105	IA2	CH104	IA1	CH106	CH103
**w053.31**	>50	>50	>50	>50	>50	>50	2.38	2.06	1.06	1.09	0.43	1.68	1.24
**w053.32**	>50	>50	>50	>50	>50	>50	22.8	13.89	7.53	6.29	6.09	10.78	6.76
**w053.6**	>50	>50	>50	>50	>50	>50	>50	>50	19.43	19	16.67	12.57	11.88
**w053.8**	>50	>50	>50	>50	>50	>50	>50	34.75	21.53	17.15	16.78	20.92	16.01
**w053.9**	>50	>50	>50	>50	>50	>50	>50	>50	31.44	32.21	22.7	34.57	28.81
**w078.1**	>50	>50	>50	>50	>50	>50	7.06	4.07	1.3	1.68	3.31	4.63	2.69
**w078.10**	>50	>50	>50	>50	>50	>50	15.43	8.67	4.42	6.1	3.04	5.49	7.08
**w078.14**	>50	>50	>50	>50	>50	>50	28.34	17.7	10.25	9.25	6.58	17.81	11.33
**w078.15**	>50	>50	>50	>50	21.97	49.04	2.97	2.55	0.76	0.96	0.25	2.08	1.51
**w078.16**	>50	>50	>50	>50	>50	>50	>50	>50	>50	>50	>50	>50	12.73
**w078.17**	>50	>50	>50	>50	>50	>50	>50	30.44	16.62	14.81	9.56	26.32	15.23
**w078.25**	>50	>50	>50	>50	>50	>50	30.45	10.5	21.4	22.67	29.85	15.19	17.72
**w078.3**	>50	>50	>50	>50	>50	>50	7.14	5.34	2.28	3.11	1.41	5.17	3.74
**w078.33**	>50	>50	>50	>50	>50	>50	>50	28.41	12.23	11.06	15.79	12.2	4.13
**w078.38**	>50	>50	>50	>50	>50	>50	33.25	15.49	13.81	12.51	13.26	12.85	12.32
**w078.4**	>50	>50	>50	>50	>50	>50	>50	24.76	19.03	10.56	23.86	29.17	22.48
**w078.5**	>50	>50	>50	>50	>50	>50	>50	>50	>50	>50	>50	>50	10.9
**w078.6**	>50	>50	>50	>50	>50	>50	>50	>50	22.34	23.56	20.68	17.31	3.8
**w078.7**	>50	>50	>50	>50	>50	>50	43.42	18.53	2.58	4.4	6.67	15.23	6.79
**w078.8**	>50	>50	>50	>50	>50	>50	17.56	9.7	6.07	4.85	5.27	9.33	6.41
**w078.9**	>50	>50	>50	>50	>50	>50	47.83	15.44	13.28	17.02	16.17	14	17.32
**w100.A10**	>50	>50	>50	>50	>50	>50	>50	48.74	25.12	21.8	27.54	37.29	36.75
**w100.A11**	>50	>50	>50	>50	>50	>50	>50	12.24	4.13	3.13	2.11	3.99	10.74
**w100.A12**	>50	>50	>50	>50	>50	>50	33.48	20.53	13.17	10.77	5.98	17.72	18.02
**w100.A13**	>50	>50	>50	>50	>50	>50	>50	17.24	3.96	3.49	10.36	6.63	26.34
**w100.A2**	>50	>50	>50	>50	>50	>50	>50	25.34	4.85	7.63	3.42	9.21	21.36
**w100.A3**	>50	>50	>50	>50	>50	>50	13.27	7.42	2.87	3.81	1.02	6.1	5.76
**w100.A4**	>50	>50	>50	>50	>50	>50	>50	25.45	12.83	10.51	12.93	14.06	13.65
**w100.A5**	>50	>50	>50	>50	>50	>50	>50	20.69	3.7	3.34	1.85	5.64	15.63

Table S5. *Cont.*

gp160	UCA	IA8	IA7	IA6	IA5	IA4	IA3	CH105	IA2	CH104	IA1	CH106	CH103
**w100.A6**	>50	>50	>50	>50	>50	>50	16.02	12.64	5.79	5.27	6.34	12.01	11.51
**w100.B3**	>50	>50	>50	>50	>50	>50	>50	12.56	3.18	3.42	2.06	4.64	10.72
**w100.B4**	>50	>50	>50	>50	>50	>50	1.64	0.75	1.13	0.64	0.91	0.74	3.46
**w100.B6**	>50	>50	>50	>50	>50	>50	>50	13.02	2.77	3	3.15	3.41	10.97
**w100.B7**	>50	>50	>50	>50	>50	>50	41.77	14.31	4.89	3.66	2.38	5.38	11.33
**w100.B8**	>50	>50	>50	>50	>50	>50	>50	>50	>50	>50	>50	>50	44.9
